# Hydralazine inhibits cysteamine dioxygenase to treat preeclampsia and senesce glioblastoma

**DOI:** 10.1126/sciadv.adx7687

**Published:** 2025-10-15

**Authors:** Kyosuke Shishikura, Jiasong Li, Yiming Chen, Nate R. McKnight, Thomas P. Keeley, Katelyn A. Bustin, Eric W. Barr, Snehil R. Chilkamari, Mahaa Ayub, Sun Woo Kim, Zongtao Lin, Ren-Ming Hu, Kelly Hicks, Xie Wang, Donald M. O’Rourke, J. Martin Bollinger, Zev A. Binder, William H. Parsons, Kirill A. Martemyanov, Aimin Liu, Megan L. Matthews

**Affiliations:** ^1^ Department of Chemistry, University of Pennsylvania, Philadelphia, PA, USA.; ^2^ Department of Chemistry, The University of Texas at San Antonio, San Antonio, TX, USA.; ^3^ Department of Neuroscience, The Herbert Wertheim UF Scripps Institute for Biomedical Innovation & Technology, University of Florida, Jupiter, FL, USA.; ^4^ Department of Physiology, Anatomy & Genetics, Target Discovery Institute, and Ludwig Institute for Cancer Research, Nuffield Department of Medicine, University of Oxford, Oxford, UK.; ^5^ Cardiovascular Institute, Perelman School of Medicine, University of Pennsylvania, Philadelphia, PA, USA.; ^6^ Sidney Kimmel Medical College, Thomas Jefferson University, Philadelphia, PA, USA.; ^7^ Department of Neurosurgery, Perelman School of Medicine, and Glioblastoma Translational Center of Excellence, Abramson Cancer Center, University of Pennsylvania, Philadelphia, PA, USA.; ^8^ Department of Chemistry and Biochemistry and Molecular Biology, The Pennsylvania State University, State College, PA, USA.; ^9^ Department of Chemistry and Biochemistry, Oberlin College, Oberlin, OH, USA.

## Abstract

Hydralazine (HYZ), a treatment for preeclampsia and hypertensive crisis, is listed by the World Health Organization as an essential medicine. Its mode of action has remained unknown through its seven decades of clinical use. Here, we identify 2-aminoethanethiol dioxygenase (ADO), a key mediator of targeted protein degradation, as a selective HYZ target. The drug chelates ADO’s metallocofactor and can alkylate one of its ligands. The resultant inactivation stabilizes regulators of G protein signaling (RGS4 and RGS5) that ADO normally marks for proteolysis, explaining the drug’s vasodilatory activity and comporting with observations of diminished RGS levels in both clinical preeclampsia and a mouse model thereof. Its inhibition of ADO suggested use of HYZ against glioblastoma (GBM); indeed, a single dose robustly senesces cultured GBM cells. By establishing ADO as a nexus for GBM and preeclampsia and connecting it to HYZ, the results create opportunities for directed tailoring of the old drug for new therapies.

## Teaser

The decades-old preeclampsia drug hydralazine vasodilates and senesces glioblastoma cells via the same enzyme target, ADO.

## INTRODUCTION

Before the 1980s, most drugs were found by in vivo testing and clinical observation (*1*). Consequently, the targets and mechanisms of action of many enduring drug therapies remain unknown. Post hoc target identification, often called “deorphanization,” sets the stage for the use of modern chemical biology tools to improve a drug by increasing its target avidity or narrowing its target profile. Moreover, if it happens that the identified target has roles in other disease states, then deorphanization of a drug can immediately provide a clinic-ready lead compound for these other diseases and can enable fast-tracked repurposing for previously unknown indications.

Hydralazine (HYZ; Apresoline) is one of the oldest Food and Drug Administration (FDA)–approved vasodilators. Originally developed as a treatment for malaria, it was introduced into the clinic more than 70 years ago. The potent and direct vasodilatory activity of HYZ has secured its place on the World Health Organization’s list of essential medicines (*2*) for the clinical treatment of hypertensive crisis (*3*) and (pre)eclampsia (*3*, *4*), a hypertension-associated obstetric emergency that is the leading cause of maternal and fetal mortality. However, it has been supplanted by newer drugs in most of its other original indications (e.g., subacute hypertension). The primary reason is that HYZ has both unfavorable pharmacokinetics and serious side effects, including, for example, a lupus-like autoimmune syndrome, blood disorders, and nerve and liver toxicity. These serious side effects are likely to arise from the drug’s engagement of multiple targets. Structure-activity studies to increase its target specificity have not been possible because the primary target mediating the desired vasodilatory effect (e.g., HYZ’s mechanism of action) remains unknown.

Vasodilation is an adaptive physiological response for immediate and preferential delivery of oxygen and essential nutrients to microenvironments, consuming them at rates exceeding their delivery. When blood flow is impaired by increased vascular resistance of maladaptive vessels, blood pressure increases, and hypertension can ensue. Dynamic constriction and dilation of vessels are modulated by changes in calcium (Ca^2+^) ion concentrations within vascular smooth muscle cells. Hence, both normal and pharmacologically induced vasodilations result from decreasing intracellular Ca^2+^ levels. The specific mechanisms to achieve this effect are well established in general and vary for the different classes of vasodilators (*5*). For the case of HYZ, however, they remain unknown.

To date, a number of potential targets for HYZ have been proposed. They include prolyl hydroxylase domain (PHD) enzymes (*6*), the Keap1-Nrf2 complex (*7*), aldehyde oxidase (*8*), protein kinase A (*9*), and glutamine oxaloacetate transaminase 1 (*10*). Supporting evidence has consisted of either observations of downstream physiological effects of the sort expected for inhibition of the proposed target or demonstration of the inhibition of that enzyme in vitro. In no case have both lines of evidence been provided to directly connect the engagement of the target by the drug to the cellular and physiological effects of its inhibition. Moreover, no links between inhibition and the effects on Ca^2+^ levels that one would expect to drive vasodilation have been established.

**Fig. 1.**
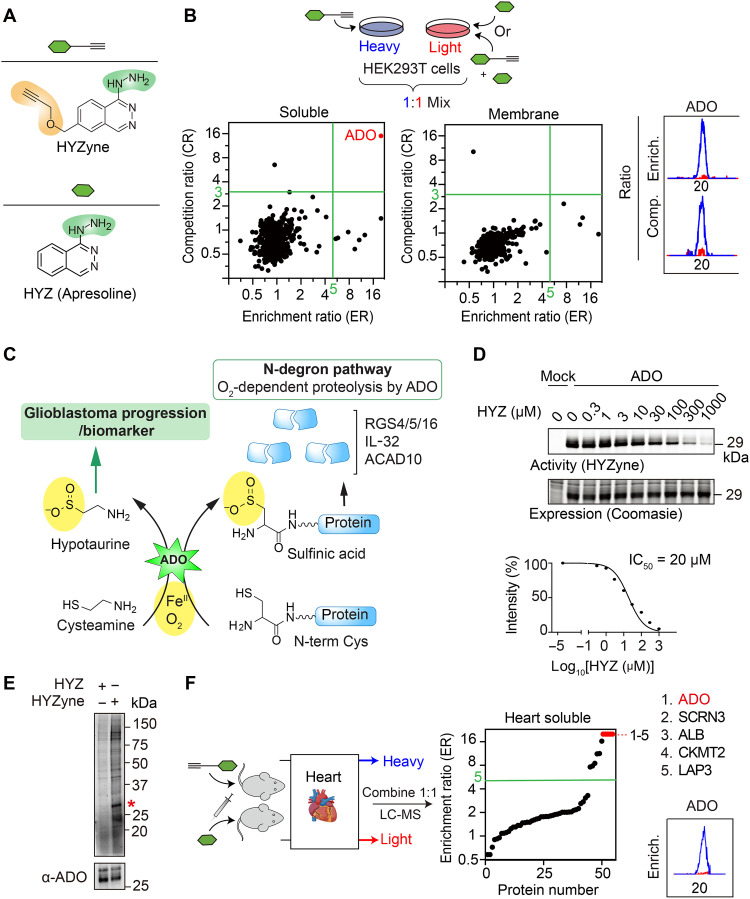
Identification of protein targets of HYZ. (**A**) Structures of HYZ drug and corresponding alkynylated drug probe (HYZyne). Hydrazine pharmacophores are shown in green, and chemical modifications to incorporate the clickable alkyne handle are shown in orange. (**B**) Schematic (top) for enrichment and competition quantitative proteomics experiments as described in the text. Isotopically heavy and light cells, proteomes, and peptides are depicted in blue and red, respectively. Quadrant plot of average enrichment [HYZyne versus HYZ (1 mM for 0.5 hours)] versus competition [HYZyne versus HYZyne pretreated with 10× HYZ (10 mM for 0.25 hours)] protein ratios. HYZyne-targeted proteins (in top right quadrants) from soluble (left) and membrane (middle) proteomes of human embryonic kidney (HEK) 293T cells are highlighted in red. Extracted parent ion chromatograms and corresponding heavy/light ratios for representative tryptic peptides of the only protein target in both experiments. Comp., competition; Enrich., enrichment. (**C**) Schematic of catalytic machinery and function of ADO. ADO uses Fe(II) and O_2_ to oxidize thiol groups of metabolic (cysteamine) and protein (N*-*terminal Cys residues) substrates. The latter serves as a critical N-degron recognition element for targeted degradation. N-term, N-terminal. (**D**) Gel-based profiles for HYZyne labeling (1 mM for 0.5 hours; top) and corresponding expression (Coomassie staining; bottom) in ADO-overexpressing HEK293T cells. Labeling is blocked by pretreatment with HYZ (for 0.5 hours) in a concentration-dependent manner, affording a median inhibitory concentration (IC_50_) value for inhibiting ADO activity in cells overexpressing the protein (plot of diminution in band intensity; bottom). (**E**) HYZyne labeling (top) and endogenous ADO expression (bottom) in nontransfected cells treated with HYZyne at approximately the IC_50_ concentration determined for HYZ above (10 μM for 0.5 hours). Asterisk (*) highlights a HYZyne-reactive protein that corresponds to the molecular weight of ADO. (**F**) Quadrant plot of average enrichment reductive dimethylation (ReDiMe) ratios versus protein number for HYZyne [4 hours, 50 mg/kg, intraperitoneally (ip)] from quantitative proteomics experiments in the soluble proteomes of mouse heart. SCRN3, secernin 3; ALB, albumin; CKMT2, mitochondrial creatine kinase 2; LAP3, leucine aminopeptidase 3.

In prior studies, we used organohydrazines (–NHNH_2_) resembling those found in HYZ as covalent probes to profile enzymes with electron-deficient cofactors (*11*–*15*). For most enzymes that we identified, the hydrazine pharmacophore was lost (most likely as N_2_), and the N-bonded carbon of the probe became coupled to an amino acid side chain near the initiating cofactor (fig. S1). Anticipating that HYZ could potentially react with electron-deficient catalytic cofactors in a similar manner, we deployed our previously reported probe, HYZyne (Fig. 1A) (*13*), which has the HYZ substructure and an additional remote alkyne group to allow for covalent azide capture of the probe together with any target to which it covalently appends, in an effort to identify the drug’s primary therapeutic target.

## RESULTS

### HYZ selectively targets 2-aminoethanethiol dioxygenase in cells and tissues

Treatment of human embryonic kidney (HEK) 293T cells with HYZyne (0.25 to 5 mM for 0.5 hours) resulted in the concentration-dependent labeling of multiple proteins (fig. S2A), as visualized by SDS–polyacrylamide gel electrophoresis (SDS-PAGE) and in-gel fluorescent scanning (*13*). Tagging of cellular proteins with 1 mM HYZyne for 0.5 hours was blocked by pretreatment with a 10-fold excess of HYZ (10 mM for 0.25 hours; fig. S2B), and band intensities uncorrelated with levels of protein expression (fig. S2, A and B). We identified high-occupancy targets of the probe by use of well-established quantitative mass spectrometry (MS)–based proteomics methods (*16*). In contrast to our other hydrazine-containing probes that identified more than 100 high-occupancy targets (externally validated) in the same cell line (*12*), we found HYZyne to be unexpectedly selective for a single high-occupancy enzyme target, 2-aminoethanethiol (cysteamine) dioxygenase (ADO; Fig. 1B and data S1). ADO, a ferrous ion [Fe(II)]–dependent thiol dioxygenase, uses O_2_ to oxidize both the namesake small-molecule and cysteine-derived thiol groups in proteins, as elaborated below (Fig. 1C) (*17*, *18*).

Because HYZ is also effective in preclinical mouse models of hypertension (*19*), we repeated the same target identification experiments in two mouse cell lines [RAW 264.7 macrophage and Neuro 2a neuroblastoma cells (fig. S3 and data S2)]. Consistent with the HYZyne target profile from HEK293T cells, mouse ADO was also identified as a primary, high-reactivity, and high-stoichiometry target of HYZyne in both mouse cell types (fig. S3 and data S2). We verified that overexpression of ADO in the HEK293T cells allowed for visualization of the probe-captured ADO by gel-based profiling. In this analysis, pretreatment of the overexpressing cells with HYZ (for 0.5 hours) diminished the stoichiometry of HYZyne capture in a manner dependent on the HYZ concentration used in the preincubation. Quantitative analysis of the fluorescence intensity of the ADO-associated (29 kDa) band dependent on [HYZ] furnished an estimate of ~20 μM for the median inhibitory concentration (IC_50_) of HYZ for ADO (Fig. 1D). In experiments with HYZyne concentrations even less than this estimated IC_50_ (10 μM), a band at the molecular weight of ADO was detected even in wild-type (WT) HEK293T cells, after the conditions for the click capture of the alkyne handle were optimized (10-fold less rhodamine azide; Fig. 1E). ADO was detected at the same position by Western blot analysis using antibodies against ADO (Fig. 1E). The simplest interpretation is that the detected band reflects capture of endogenous ADO, although it remains possible that the band arises from one or more other proteins of similar molecular weight.

As the heart is a vital organ of the cardiovascular system and HYZ is FDA approved to treat heart failure (*3*), HYZ/HYZyne was systemically administered to WT mice to directly identify HYZ-targeted proteins in cardiac tissue. Protein target enrichment was determined by comparing heart tissue proteomes from HYZyne-treated [50 mg/kg, intraperitoneally (ip), for 4 hours] to those from HYZ-treated (50 mg/kg, ip, for 4 hours) control mice. HYZyne-labeled targets were then identified using known quantitative proteomics methods as previously described (*15*, *20*). The data confirm that ADO is indeed an enriched protein target of HYZ in heart tissue following systemic delivery to the animal (Fig. 1F, fig. S4, and data S2). Thus, ADO is a prevailing target of HYZ across native biological systems tested including murine heart, and our global proteome-wide profiling data furnished ADO as the most likely pharmacological target of HYZ. Consistent with this proposed pharmacological mechanism of action, a missense mutation in the human *ADO* gene (rs10995311: C to G; P39A) is associated with decreased blood pressure (*21*, *22*), supporting our hypothesis that ADO is the drug’s primary therapeutic target. The observations and analysis imply that the primary physiological function of ADO may lie in the regulation of vascular tension and blood pressure.

### Cofactor coordination confers selective inactivation by HYZ

**Fig. 2.**
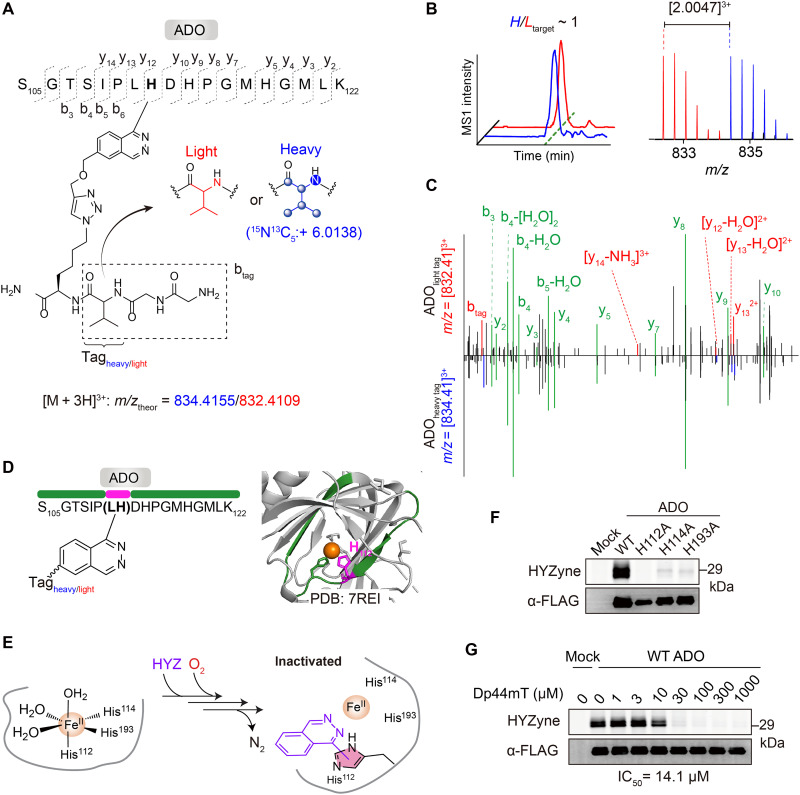
Covalent modification of active site histidine residues in ADO. (**A**) Structures and theoretical parent masses of heavy- and light-tagged ADO peptides labeled by HYZyne (1 mM for 0.5 hours) and processed by the isoTOP-ABPP (isotopic tandem orthogonal proteolysis–activity-based protein profiling) method (*23*). *m/z*, mass/charge ratio. (**B**) Extracted triply charged MS1 ion chromatograms (left) and corresponding isotopic envelopes (right) for coeluting heavy- and light-tagged peptides labeled by HYZyne (blue and red, respectively). (**C**) Comparison of high-resolution MS2 spectra for the ADO isotopic peptide pair in (B). Unshifted ions are shown in green. (**D**) Labeled peptide in the ADO crystal structure [Protein Data Bank (PDB): 7REI]. The identity of the HYZyne-containing peptide (amino acids 105 to 122) and corresponding site of labeling (resolved between Leu^111^ and His^112^) are shown in green and magenta, respectively. Metal is colored orange. (**E**) Reaction scheme and MS result–based prediction of the resultant HYZ-ADO adduct in cells. (**F**) Activity (top) and expression profiles (bottom) for HYZyne labeling of cells (100 μM for 0.5 hours) overexpressing WT ADO versus (F) catalytically inactive variants or (**G**) pretreatment with iron chelator [di-2-pyridylketone-4,4-dimethyl-3-thiosemicarbazone (Dp44mT) for 1 hour], demonstrating dose-dependent inhibition of Fe(II)•ADO at the indicated IC_50_.

To define the site and reaction manifold for covalent capture of ADO by HYZ, we isolated ADO-specific peptide(s) modified by HYZyne from cells. This chemoproteomic approach is well established (*12*, *23*) and leverages isotopically differentiated, protease-cleavable biotin-azide tags to enrich and release probe-labeled peptides that migrate and coelute as mass-differentiated pairs (Fig. 2, A and B). Among seven ADO-specific peptides that were identified with comparable parent ion (MS1) intensities, only one contained a residue (His^112^) known to be essential for enzyme activity (table S1) (*24*–*26*). This peptide pair was manually and computationally assigned as a triply charged peptide comprising residues 105 to 122 of ADO according to the high-resolution MS2 spectra of assigned y- and b-ions (Fig. 2C). Identification of the y_12_- and y_10_-ions resolved the site of probe capture between Leu^111^ and His^112^ (Fig. 2C), where His^112^ is one of ADO’s three cofactor ligand side chains that bind the enzyme’s metal center, a Fe(II), and are essential for catalysis (Fig. 2D, fig. S5, and table S1) (*24*–*26*). The parent masses for the His^112^-containing peptide pair confer the radical manifold (*12*), as they were detected within ~1–part per million error of the predicted product mass for arylation of the His^112^-coordinated side chain, presumably with the loss of hydrazine (–NHNH_2_) in the coupling event (Fig. 2E, fig. S5, and table S1).

To determine whether HYZ action requires active, functional ADO, we tested inactive variant proteins harboring substitutions of cofactor ligands for HYZyne labeling. Probe capture was abolished in the H112A, H114A, and H193A variants (Fig. 2F). In contrast, variants with substitution of Cys residues (Cys^18^, Cys^132^, Cys^183^, Cys^220^, Cys^249^, and Cys^258^) identified as other HYZyne-modified sites in the chemoproteomics analysis (table S1) did not affect the band intensity of HYZyne-labeled ADO to a detectable level (fig. S6). These data confirm that there are multiple substoichiometric labeling events (e.g., of the Cys residues) that do not result in profound inhibition but are sufficient to generate modified tryptic peptides detected by MS. Lastly, pretreating cells with di-2-pyridylketone-4,4-dimethyl-3-thiosemicarbazone (Dp44mT) (0 to 1000 μM for 1 hour), an established Fe(II) chelator with reported specificity for Fe(II)•ADO (*27*) that has structural homology to HYZ, abolished HYZyne labeling in a concentration-dependent manner and inhibited ADO with a potency in situ (IC_50_ ~ 14 μM; Fig. 2G and fig. S7) comparable to that of HYZ. Collectively, the data suggest that HYZ is an enzyme-activated irreversible inhibitor (e.g., a mechanism-based inhibitor) of ADO. Covalent modification of the active site His^112^ residue by HYZ is selective and specific for the active state of the holoenzyme [Fe(II)•ADO]. Hence, HYZyne is also a fully functional and validated activity-based protein profiling (ABPP) probe (*11*, *12*, *16*) for ADO that can be used in the future to investigate how ADO activity is (dys)regulated across diverse biological contexts [for example, upon acute changes within a (pre/sub)hypoxic cellular environment] (*27*, *28*).

**Fig. 3.**
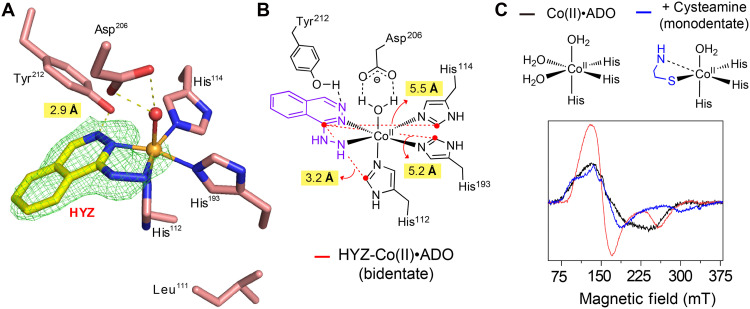
Determination of cocrystal structures of human ADO and HYZ. (**A**) Active site architecture of Co(II)•ADO. The x-ray crystal structure of the HYZ-bound Co(II)•ADO complex was determined at 1.88-Å resolution. The *F*_o_-*F*_c_ maps are contoured at 3.0 σ and colored in green. (**B**) A two-dimensional diagram of the active site showing the distances between HYZ and the three histidine ligands that coordinate the metal center. (**C**) Active site ligand environment (top) of Co(II)•ADO (black) and binding of primary substrate, cysteamine (blue). Potential interaction between Co(II) and the C_2_ amine group of cysteamine is indicated by a dashed line, suggesting a monodentate binding mode. Corresponding electron paramagnetic resonance (EPR) spectra (bottom) generated for Co(II)•ADO (300 μM) in the absence (black) or presence of excess (1 mM) cysteamine (blue) or HYZ (red) under anaerobic conditions. EPR measurements involved four scans at 3.17-mW microwave power at 30 K.

The HYZyne-ADO adduct isolated from cells has lost the hydrazine moiety of the probe. The most likely mechanisms for the implied C-N fragmentation involve nitrogen-centered radical generation. Thus, the initial step is likely to involve coordination to and activation by the metal center, analogously to the mechanism by which the substrate is oxidized. To capture the anticipated initial state of the HYZ•ADO reaction in vitro, we first purified and crystallized the Fe(II)•ADO complex as previously described (*25*). Soaking the crystals with excess HYZ resulted in the attachment of a single HYZ molecule to Trp^257^ on the protein surface, as revealed by a structure solved at 2.39-Å resolution (figs. S8A and S9, A to C, and table S2). In this structure, the Fe(II) center retained two water molecules—one fewer than in the resting Fe(II)•ADO structure (fig. S10A). Thus, the Fe(II) crystals did not capture the expected prereaction complex. Because the catalytically competent cobalt-substituted enzyme [Co(II)•ADO] has been shown to accommodate substrate binding (*26*), we next used this analog to visualize the initial interaction. Soaking the Co(II)•ADO crystals with excess HYZ resulted in the formation of the HYZ•Co(II)•ADO complex, as revealed by a structure solved to a resolution of 1.88 Å. Electron density for the parent drug of HYZ was clear near the active site metal center (Fig. 3A), while a second molecule was bound to the protein surface (figs. S8B and S9, D to I, and table S2) as for the Fe(II)•ADO complex structure.

As expected, HYZ binds in a bidentate chelating mode to the same site as the primary substrate, replacing two H_2_O molecules coordinated by the metal center (fig. S10B). In addition, a hydrogen bond between Tyr^212^ and the N3 atom of the phthalazine ring creates a pseudo-tridentate situation, underscoring the critical role of both the phthalazine ring’s amine groups (N2 and N3) and the hydrazine moiety. These features likely represent nonmodifiable elements in designing future ADO inhibitors that exploit this mechanism. Notably, the third H_2_O ligand in the equatorial position remains bound, stabilized by a hydrogen bond with the carboxylate group of Asp^206^. This structural evidence supports the classification of HYZ as a competitive inhibitor that preserves the enzyme’s capacity to bind and activate O_2_, analogous to its interaction with endogenous substrate (*29*).

Further structural analysis revealed that His^112^ is significantly closer to HYZ (3.2 Å) compared to other ligands including His^114^ and His^193^ (5.5 and 5.2 Å, respectively) (Fig. 3B), whereas Leu^111^, which we could not resolve by MS alone, projects away from the active site (Fig. 3A). The data confirm covalent modification (alkylation) of His^112^ from both our in situ and in vivo chemoproteomic assays (Fig. 2E). Last, structural alignment of Co(II)•ADO crystallized in the absence and presence of HYZ confirms that HYZ binding does not perturb the active site architecture (fig. S10B).

To further investigate the binding dynamics, we used electron paramagnetic resonance (EPR) spectroscopy to compare the electronic environments of the EPR-active Co(II)•ADO, which has an unpaired d^7^ electron configuration expected to be differentially perturbed when bound to no substrate (black trace; Fig. 3C), with cysteamine (blue trace) versus HYZ (red trace). It has been proposed that, under certain conditions, cysteamine binds in monodentate fashion via its thiol group (*29*), causing a shift in g-tensor values of the unpaired electron spin, which reflects a change in the metal coordination environment upon substrate binding. In contrast, the EPR spectrum of HYZ•Co(II)•ADO is more markedly perturbed, distinct from both resting Co(II)•ADO and cysteamine•Co(II)•ADO and consistent with HYZ binding in a bidentate fashion (Fig. 3C and table S3).

As the observed covalent capture of ADO by the HYZyne in cells and animals was not recapitulated under the conditions used for our spectroscopic and structural studies, we sought to further investigate the reaction in vitro by continuous monitoring of O_2_ consumption under multiple-turnover conditions by Fe(II)•ADO in the absence and presence of HYZ. Kinetic analysis, illustrated in a Lineweaver-Burk plot (fig. S11A), revealed a competitive inhibition pattern. Although the reciprocal plots do not converge perfectly at the *y* axis, the deviation is minimal, and the *K*_i_ values calculated by global fitting of the double reciprocal plots were 2.48 ± 0.42 and 13.88 ± 7.51 μM, respectively. We were unable to identify assay conditions leading to time-dependent inactivation, as one would expect to observe with a mechanism-based covalent inactivator (fig. S11B). Hence, these in vitro experimental results support HYZ’s predominant role as a competitive inhibitor, and the reported *K*_i_ reflects only the initial, reversible binding event (*30*). Therefore, the enzyme-activated irreversible inhibition reaction observed in vivo likely reflects a distinct, mechanism-based process not yet captured by the in vitro system.

### Accumulation of RGS4 and RGS5 upon ADO inhibition by HYZ

ADO is an evolutionarily conserved iron-dependent thiol dioxygenase and enzymatic oxygen sensor that serves as the cell’s immediate frontline responder, transducing (sub)acute changes in O_2_ tension. By simultaneously rewiring its metabolism in the cytoplasm, it also deploys long-range alert signals in an expedited fashion, bypassing both timelines and resources required by transcriptional and translational machinery for protein production. As the lowest affinity (highest *K*_m_ for O_2_) oxygen-dependent enzyme known to date (*18*, *28*), ADO converts the metabolite cysteamine to hypotaurine in cells. Furthermore, ADO also transduces targeted oxygen-dependent degradation of important regulatory proteins with N-terminal cysteine residues, following removal of genetically encoded N termini (Fig. 1C). HYZ prevents formation of the N-degron recognition element—oxidation of N-Cys-SH groups to their corresponding sulfinic acids (–SO_2_^−^) (Fig. 1C)—an essential posttranslational modification required for targeted protein degradation by the ubiquitin-proteasome system (*31*). In the subsequent step, arginyltransferases (e.g., ATE1) append this destabilizing residue (l-Arg) to the oxidized *N terminus*, thereby promoting its degradation (fig. S12). Known N-degron substrates of ADO include the following: (i) three regulators of G protein signaling (RGS4, RGS5, and RGS16), which are guanosine triphosphatase (GTPase)–activating proteins for heterotrimeric G proteins that control the duration and extent of G protein–coupled receptor (GPCR) signaling (*31**–**33*); (ii) the atypical angiogenic cytokine interleukin-32 (IL-32) (*18*); and (iii) although not as-of-yet extensively demonstrated by comparison, brain-specific metabolic enzyme acyl–coenzyme A dehydrogenase family member 10 (ACAD10), which is central to β-oxidation of fatty acids in the mitochondria (*34*).

**Fig. 4.**
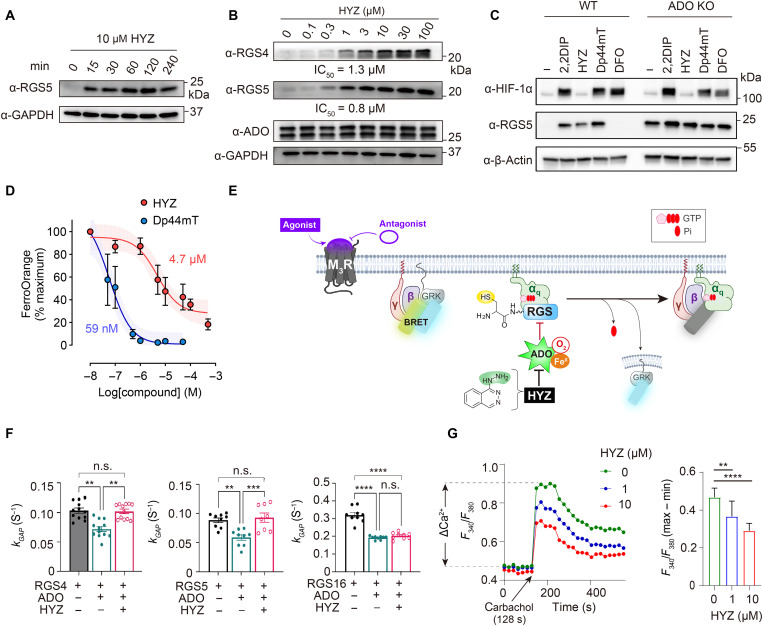
HYZ controls the ADO-dependent N-degron pathway and GPCR signaling in cells. (**A**) Time- and (**B**) concentration-dependent changes in endogenous levels of respective RGS substrates and control proteins as a function of HYZ (10 and 0 to 100 μM for 1 hour, respectively) in SH-SY5Y cells, as determined by Western blot. (**C**) Endogenous HIF-1α, RGS5, and β-actin protein levels in WT or *ADO*-deficient SH-SY5Y cells treated with HYZ (10 μM) or iron chelators [2,2-dipyridyl (2,2DIP; 100 μM), HYZ, Dp44mT (10 μM), or DFO (250 μM)] for 4 hours. KO, knockout. (**D**) Intracellular labile Fe(II) levels determined by FerroOrange in SH-SY5Y cells treated with HYZ or Dp44mT at the indicated concentrations (4 hours). Data are represented as mean ± SEM (*n* = 3 independent experiments). (**E**) Schematic diagram of the bioluminescence resonance energy transfer (BRET) assay. The rate of G protein deactivation is reflected as the rate of decrease in BRET signal. Agonist (acetylcholine) and antagonist (atropine) were used to activate and deactivate muscarinic 3 receptor (M_3_R), respectively. [Part of (E) created in BioRender; K. Shishikura (2025) (https://BioRender.com/nm7ebk3)]. Pi, inorganic phosphate; (**F**) BRET assay assessing the effect of HYZ (200 μM) on *k*_GAP_ activity of RGS4, RGS5, and RGS16 in the presence or absence of ADO. Data are represented as mean ± SEM (*n* = 3 independent experiments, each performed with three replicates). One-way analysis of variance (ANOVA) with Tukey’s multiple comparisons test; **P < 0.005, ***P = 0.0008, ****P < 0.0001, and n.s. = not significant. (**G**) Ca^2+^ influx upon M_3_R activation with carbamoylcholine (carbachol; 100 μM) in Fura-2–loaded SH-SY5Y cells following HYZ treatment (0 to 100 μM for 1 hour). Average traces of F_340_/F_380_ (left) and quantification of the maximum increase in Ca^2+^ (right) in response to carbachol (128 s; arrow) normalized to minimum baseline Ca^2+^ levels. Data are represented as mean ± SD (*n* = 7 to 8 replicates). **P < 0.01 and ****P < 0.0001; one-way ANOVA with Tukey’s post hoc test.

To further test the impact of HYZ on ADO’s activity at the cellular level, we treated SH-SY5Y cells, a human neuroblastoma cell line commonly used for neuroscience research and previously used to study the impact of genetic deletion of *ADO* (*18*, *27*). Among ADO substrates, we initially focused on RGS4 and RGS5 due to their established role in GPCR-mediated Ca^2+^ signaling and their clear connection to vasodilation (*35*, *36*). Accordingly, in the absence of HYZ, RGS5 protein was undetectable, consistent with its transient expression and immediate ADO-catalyzed oxidation and subsequent degradation. In contrast, in the presence of HYZ (10 μM), RGS5 protein levels increased in <1 hour, which is consistent with the immediate blood-pressure–lowering effect observed in patients following administration of the drug (Fig. 4A) (*3*, *5*). HYZ inhibited targeted degradation of RGS5, with 1 μM drug giving half the maximal protein level and 10 μM giving maximal levels (after an incubation time of 1 hour; Fig. 4B and fig. S13). These results indicate that the pharmacological action of HYZ involves stable accumulation of RGS5.

RGS4 protein levels were similarly up-regulated by HYZ, with ~1 μM drug required to give half the maximum level (Fig. 4B and fig. S13). Notably, the effects of HYZ were specific to RGS proteins, as we did not observe any changes in ADO (Fig. 4B). To determine whether the phenomenon was conserved across cell lines, we applied HYZ to HEK293T cells. Although endogenous levels of RGS4 and RGS5 fell below the limit of detection, RGS16, another known substrate, was up-regulated in response to HYZ (fig. S14A) (*31*, *33*). To enable detection, we observed stabilization of recombinant RGS5 in the presence of HYZ when cells were cotransfected to overexpress both enzyme and substrate proteins (fig. S14, B and C). These data suggest that the pharmacological effects of HYZ may be conserved across cell types.

In addition to O_2_-sensing enzymes such as ADO and among previously hypothesized targets of HYZ found in the literature (*6*), PHD enzymes also serve as O_2_ sensors, operating in concert with and having sensitivity second to that of ADO (second-highest *K*_m_ for O_2_) (*28*). They too operate on protein substrates to induce targeted degradation but instead via catalyzing a different posttranslational oxidation reaction—hydroxylation of specific proline residues found internally at specific site(s) in protein substrates (*28*). Among them includes hypoxia-inducible transcription factor 1α (HIF-1α), the expression for which is specifically stabilized in a hypoxic environment, allowing it to translocate to the nucleus for regulation of gene expression required for metabolic adaptation, angiogenesis, and cell survival under low oxygen conditions (*27*, *28*). Given the functional similarity, we next sought to confirm that HYZ is specific for the ADO/RGS5 pathway and selective against the PHD/HIF-1α pathway. By monitoring RGS5 versus HIF-1α substrate protein levels as a function of [HYZ] and an indirect readout of ADO versus PHD activity, respectively, in SH-SY5Y cells (analogous to Fig. 4B), HYZ was found to be 100-fold more selective for ADO over PHDs (fig. S15). The data are consistent with poor enrichment and absence of competition we observed for these targets in our chemoproteomic profiling experiments (Fig. 1B and fig. S3). Next, to evaluate the potency and selectivity of HYZ against established cell-permeable chelators of labile Fe(II) (*27*), we showed that both RGS5 and HIF-1α [for chelators 2,2-dipyridyl (2,2DIP) and Dp44mT] or HIF-1α alone [deferoxamine (DFO)] is altered. In contrast, HYZ selectively altered only RGS5 protein levels in an ADO-dependent manner (Fig. 4C), thereby distinguishing its mechanism from that of conventional Fe(II) chelators, which have been shown most frequently throughout the literature to affect HIF-1α protein levels (via PHD enzymes) (*27*). Comparison of band intensities for stabilized RGS5 in WT versus *ADO*-deficient cells confers HYZ selectivity for ADO and validates that RGS5 stabilization is independent from activity states of PHD enzymes (Fig. 4C). Selectivity for ADO is consistent with the observation of HYZ’s 80-fold weaker binding affinity toward labile Fe(II) pools within cells in comparison to Dp44mT, an established Fe(II) chelator previously shown to have enhanced potency for ADO and has structural homology with HYZ (Fig. 4D) (*27*). The data demonstrate that HYZ is the first reported specific inhibitor of ADO, exhibiting selectivity against PHD enzymes (by >100-fold) and weaker chelating affinity of labile Fe(II) compared to that of Dp44mT (by 80-fold), despite having potency for ADO comparable to that of HYZ. The data demonstrate that, not unexpected for an already FDA-approved drug, HYZ has superior druglike properties compared to known metal chelating reagents.

### HYZ-promoted RGS accumulation attenuates GPCR signaling

Given that RGS proteins attenuate GPCR signaling, the demonstration of their accumulation upon treatment with HYZ provides a plausible link for the control of Ca^2+^-mediated mobilization that drives vasodilation. Hence, we evaluated the ADO- and HYZ-dependent changes in the rate of deactivation of Gα_q_, the G protein pivotal to initiating Ca^2+^ release that RGS4, RGS5, and RGS16 are known to regulate (*37*). Using an established bioluminescence resonance energy transfer (BRET)–based assay, we quantified the GTPase activating protein (GAP) activity of RGS4, RGS5, and RGS16 by monitoring their ability to accelerate the rate of Gα_q_ reassociation with its Gβγ subunits upon termination of GPCR signaling (*k*_GAP_; Fig. 4E) (*37*). First, we found that overexpression of ADO reduced RGS4 and RGS5 GAP activity upon inactivation of the muscarinic 3 receptor (M_3_R) and that this effect was reversed by HYZ (200 μM) (Fig. 4F and fig. S16). The data indicate that HYZ indeed inhibits GPCR signaling via the ADO-RGS axis in a concentration-dependent manner (fig. S17). Notably, RGS16 GAP activity was not recovered under these conditions (Fig. 4F), suggesting that this RGS protein may be subject to other regulatory mechanisms that influence its ability to control M_3_R signaling. The complete dataset of GAP activity responses by HYZ is shown in fig. S18.

### HYZ alters mobilization of GPCR-mediated intracellular Ca^2+^

As Gα_q_-coupled receptors mediate intracellular Ca^2+^ release in vascular smooth muscle cells to maintain vascular tone and healthy blood flow, we stimulated M_3_R, the subtype predominantly expressed by SH-SY5Y cells (*38*), with a synthetic muscarine agonist, carbamoylcholine (carbachol), and measured HYZ-dependent changes in intracellular Ca^2+^ concentrations. Using Fura-2, a high-affinity ratiometric fluorescent indicator that specifically binds free Ca^2+^ ions inside cells, we showed that HYZ significantly reduced GPCR-dependent intracellular Ca^2+^ levels in a concentration-dependent manner (Fig. 4G), within the same concentration range that modulates RGS protein levels (Fig. 4B). Collectively, the results complement genetic data previously reported for *ADO* deficiency derived from the same cell line (*18*). Overall, the data explain the pharmacological activities of the drug and its molecular, cellular, and physiological mechanisms of action in evoking vasodilation for the treatment of preeclampsia.

HYZ frequently (~10% incidence) induces a lupus-like condition in patients (*39*). Lupus is an autoimmune condition that commonly manifests as skin rashes and joint pain (*39*). Its mechanism is largely unknown; however, decreased extracellular signal–regulated kinase (ERK) phosphorylation in patient T cells has been cited as a potential contributing factor (*40*–*42*). Because Gα_q_-coupled receptors mediate ERK phosphorylation (*43*) and are negatively regulated by RGS substrates (*33*), we measured HYZ-dependent changes in ERK phosphorylation under the same conditions as above. A concentration-dependent decrease in ERK phosphorylation was observed with HYZ following receptor stimulation with carbachol (fig. S19), suggesting that the drug’s lupus-like side effects may be attributed to ADO inhibition and/or reduced ADO activity in patient T cells.

### ADO inhibition by HYZ senesces glioblastoma cells

**Fig. 5.**
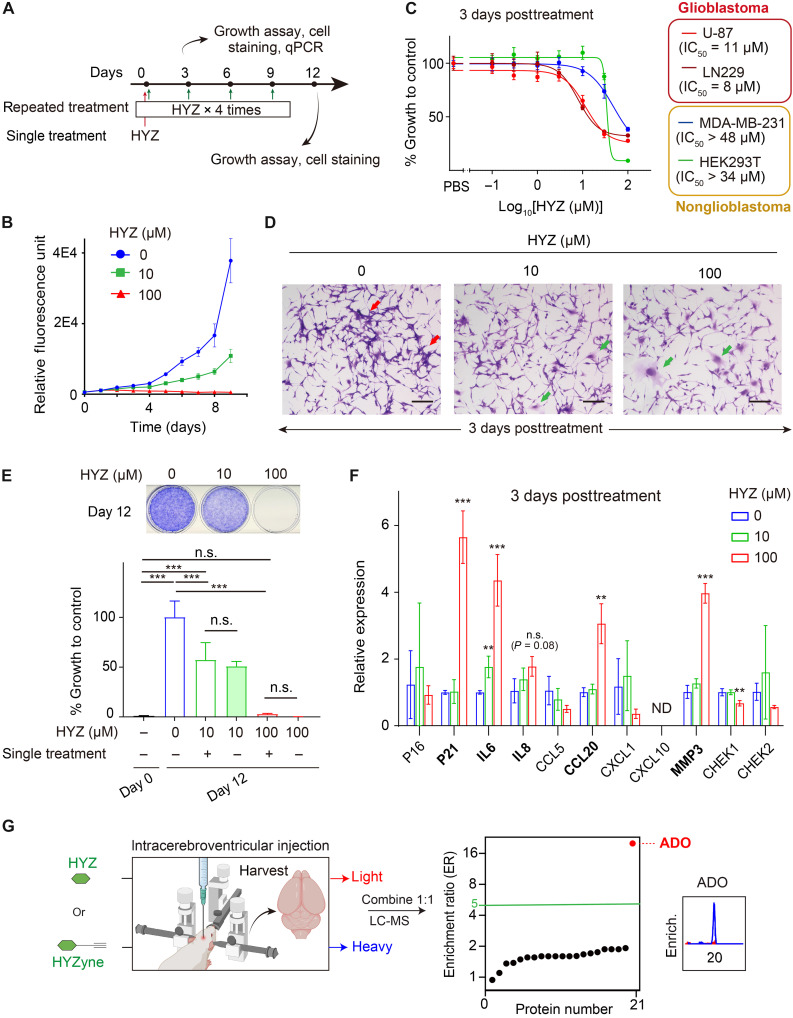
Glioblastoma growth inhibition by HYZ. (**A**) Schematic illustration of the experimental design of the study describing the HYZ treatment regimen and evaluation. (**B**) Quantification of U-87 cells over time from vehicle control and HYZ (10 or 100 μM) treatment groups measured by alamarBlue assay. (**C**) Growth of varying cell lines 3 days posttreatment of HYZ. Data are represented as mean ± SD (*n* = 5 replicates) (**D**) Representative crystal violet staining of U-87 cells obtained from indicated HYZ treatment groups (0 to 100 μM). Red arrow: clusters of U-87 cells; green arrow: enlarged U-87 cells. Scale bars, 200 μm. (**E**) Growth of U-87 cells after 12-day culture with single and repeated HYZ treatments (0 to 100 μM). Viable cells after repeated treatments are visualized by crystal violet staining (top). Viable cells under various conditions were quantified by alamarBlue assay (bottom). Data are represented as mean ± SD (*n* = 6 replicates). Statistical analysis was conducted using one-way ANOVA, followed by Tukey’s post hoc test, with pairwise comparisons made across all treatment groups. ***P < 0.001. n.s., not significant. (**F**) Quantitative reverse transcription polymerase chain reaction (qRT-PCR) analysis of indicated senescence marker gene expression in U-87 cells after 3-day culture with HYZ (0 to 100 μM). Data are represented as mean ± SD (*n* = 3 replicates). Statistical analysis was conducted using one-way ANOVA, followed by Tukey’s post hoc test, with pairwise comparisons made to the untreated control for each gene. **P < 0.01, ***P < 0.001. n.s., not significant; ND, not detected. (**G**) Quadrant plot of average enrichment ReDiMe ratios versus protein number for HYZyne from quantitative proteomics experiments in the soluble proteomes of mouse brain postintracerebroventricular injection of HYZ or HYZyne (30 μg for 4 hours). Protein targets with enrichment ratio (ER) ≥ 5 were considered high-reactivity targets and are annotated. [Part of (G) created in BioRender; K. Shishikura (2025) (https://BioRender.com/c8sf0uw)].

Elevated hypotaurine levels (the product of ADO’s metabolite substrate) (*44*) and increased ADO expression (*45*) have both been clinically linked to malignancy of glioblastoma and experimentally shown to be validated drivers of tumor growth and aggressive behavior. These data suggest that ADO is a promising target for glioblastoma, but no inhibitors for ADO have been reported to date. Given that HYZ is a more than 70-year-old drug with a well-established safety profile, we explored ADO inhibition as a potential therapeutic strategy for glioblastoma first by treating cell lines with HYZ (Fig. 5A). A single treatment (at both 10 and 100 μM) inhibited growth of U-87 cells throughout a time period of up to 9 days (Fig. 5B). Notably, chemoproteomics-based target identification experiments in this cell line and treated with the same concentration of HYZyne (100 μM) identified ADO as a high-occupancy target (fig. S20). At 3 days posttreatment, both U-87 and LN229 glioblastoma cell lines displayed concentration-dependent growth inhibition that plateaued at ~30 μM with IC_50_ values of ~10 μM (Fig. 5C). These glioblastoma cell lines were more sensitive to HYZ than noncancer (HEK293T) and breast cancer cell lines (MDA-MB-231) tested, which did not show a plateau and had estimated IC_50_ values of >30 and 50 μM, respectively. These results may suggest that HYZ has a potential general selectivity for the most aggressive cancer types, as recently reported data from The Cancer Genome Atlas, which demonstrated that *ADO* expression in liver, cervical, and pancreatic cancers is inversely correlated with patient survival (*46*, *47*). More comprehensive studies are needed to evaluate the full scope of HYZ’s anticancer activity.

Growth inhibition is represented by two general mechanisms: cytotoxic and cytostatic (*48*). We evaluated cytotoxicity at an early (6 hours) time point, when cytotoxicity is typically observed (*49*, *50*), and a later time point (24 hours), when cell growth differences begin to emerge (Fig. 5B). No significant cell death was observed between treatment groups at either time point (fig. S21), suggesting that HYZ is cytostatic rather than cytotoxic. Three days posttreatment, U-87 cells, which normally form cellular clusters, displayed morphological changes characterized by the absence of cluster formation and the presence of cellular enlargement with increasing HYZ concentrations (Fig. 5D). These features are often associated with cell-cycle arrest and cellular senescence (*51*). To investigate whether HYZ induces a senescent state, common for compounds with known cytostatic anticancer activity (*51*), we monitored cell growth over a 12-day (long-term) period following repeated 3-day treatments with HYZ (10 versus 100 μM). No substantial growth was observed at either concentration (Fig. 5E).

As cellular senescence is considered a terminal state of growth, one would therefore expect repeated or ongoing exposures to HYZ to be ineffectual with respect to cell state and growth inhibition. We explicitly evaluated this prediction, finding that there was no substantial difference at either of the HYZ concentrations tested between a single dose and repeated exposures to the drug. In other words, prolonged senescence is robustly induced by just a single HYZ treatment (Fig. 5E). In addition, 3 days following treatment of U-87 cells with HYZ, cellular mRNA expression levels of senescence marker *p21* and senescence-associated secretory phenotype (SASP) markers, including *IL6*, *IL8*, C-C motif chemokine ligand 20 (*CCL20*), and matrix metalloproteinase 3 (*MMP3*), were increased significantly (e.g., *P* < 0.05 with the exception of *IL8*; *P* = 0.08) relative to their levels in untreated cells (Fig. 5F). The extent of enhanced accumulation of these markers was greater at higher HYZ concentrations. These observations are consistent with our hypothesis that HYZ drives authentic senescence in its cytostatic activity. Among the SASP markers, plasma levels of the CCL20 protein are known to be increased in patients with glioblastoma (*45*) and even suggested to be predictive of preeclampsia (*52*). The primary structure of CCL20 has the relatively rare hallmark of an ADO substrate—a predicted Met-Cys N terminus (as originally synthesized by the ribosome), suggesting that it could be a direct physiological substrate of ADO.

### HYZ can inhibit ADO in the brain

HYZ itself has a poor blood-brain barrier (BBB) penetrance precluding delivery of the drug to the brain (*53*, *54*), thereby limiting its efficacy in treating the most severe and deadly symptoms of (pre)eclampsia (e.g.*,* seizure, coma, or cerebral hemorrhage) that can occur without warning (*55*). To ensure that HYZ can indeed engage ADO in the brain, we bypassed the BBB by delivering HYZyne versus HYZ (30 μg) to mice by intracerebroventricular injection, followed by global target analysis of brain tissue harvested from the euthanized animals. The data show that ADO is the only detectable high-enrichment target from the brain proteome (Fig. 5G). Hence, HYZyne may be used as a pharmacological tool in the future to evaluate in vivo ADO activity for disease indications including but not limited to preeclampsia and/or glioblastoma.

## DISCUSSION

The results directly connect HYZ inhibition of ADO, increased accumulation of the known ADO substrates RGS4 and RGS5, attenuation of associated GPCR-mediated cell signaling, and subsequent reduction of intracellular Ca^2+^ levels, a key driver of vasodilation. These molecular, cellular, and physiological mechanisms for the pharmacological effect of HYZ complement previous reports of diminished RGS5 levels in the placenta and maternal arteries of preeclamptic mothers (*36*). Similarly, mice with the *RGS5* gene disrupted exhibit the key characteristics of preeclampsia: hypertension, proteinuria, placental pathology, low birth weight, hyperresponsiveness to angiotensin II and glutamate signaling, BBB permeability, and stroke (*36*, *56*). Although comprehensive studies have not yet been performed, several lines of evidence also indicate that RGSs inhibit eclamptic seizures (*57*, *58*), suggesting that a brain penetrant HYZ derivative could be effective against the most life-threatening complications of the disease, which include seizure, stroke, and permanent organ damage (*59*).

The mechanism of HYZ action implicated by our results can explain existing genetic and (pre)clinical data reported for (pre)eclampsia, a condition with a known drug but unknown therapeutic target(s). Ironically, glioblastoma presents precisely the opposite case. ADO has been touted as a potential therapeutic target for this disease because its expression and the up-regulation of its metabolic product, hypotaurine, drive growth of glioblastoma cells (*44*, *45*) and are clinical predictors of tumor grade and patient outcome (*45*, *60*), as has also been seen for other cancers (*47*). However, there had been no compounds reported to effectively inhibit ADO to evaluate for therapeutic effects in glioblastoma. Our association of HYZ with its target, ADO, thus solves long-standing challenges in drug development for both diseases: It paves the way for rational modification of HYZ to improve its pharmacokinetics and target selectivity against preeclampsia, and it affords an initial, safe lead compound against ADO to be adapted for BBB penetrance for the treatment of glioblastoma.

The comparative studies with Fe(II) chelators and PHD enzymes further showed that ADO inhibition by HYZ is direct and specific to ADO and selective against PHD enzymes and labile intracellular Fe(II) pools. HYZ operates as an irreversible mechanism-based inhibitor in cells and animals. However, attempts to identify conditions that recapitulate covalent coupling to ADO in vitro were unsuccessful, and we posit that a reactant is likely to be present in vivo that is needed to oxidize the drug to initiate its decomposition. Future efforts will seek to define such component(s). Interesting possibilities include reactive oxygen or nitrogen species, such as nitric oxide, superoxide, or peroxide, which would imply a link between cellular oxidative stress and HYZ activity.

This study identifies a specific inhibitor for ADO, which, ironically, also turned out to be the longest-standing FDA-approved vasodilator. HYZyne is a validated ABPP probe useful for evaluation of ADO activity in native biological systems and for the discovery and optimization of ADO-targeting therapies. Mechanism-based inhibition represents a viable therapeutic strategy expected to be amenable to elaboration to improve pharmacological (potency and selectivity) and clinical (efficacy and safety) properties for the treatment of at least two deadly diseases, (pre)eclampsia and brain cancer, which remain unmet medical needs.

In summary, by solving the more than 70-year-old mystery regarding HYZ’s mechanism of action, we serendipitously identified a previously unknown target for preeclampsia, a potential drug to treat glioblastoma, and a broader strategy for effective repurposing and further development of novel therapies to treat these and other intractable or rare brain diseases in the future. Matching a drug to its target provides a new pharmacological tool to define the substrate scope of ADO and elucidate its function in other disease biology. These tools will guide improvement of the drug’s properties and direct new hypotheses to solve the 2400-year-old mystery of (pre)eclamptic etiology.

## MATERIALS AND METHODS

### Clickable probes and drugs

1-hydrazineyl-6-((prop-2-yn-1-yloxy)methyl)phthalazine hydrochloride (e.g., HYZyne) was synthesized and characterized as previously described (*13*) with the exception of replacement of the counterion. Originally isolated with stoichiometric trifluoroacetate (TFA) as a result of the buffer system typically used for purification, we replaced the counterion with HCl (0.1%) so as to afford the hydrochloride salt (fig. S22), which serves as a more acceptable form for use in situ and in vivo experiments. This counterion is equivalent to that found in the drug purchased commercially and used for this study: hydralazine hydrochloride (J&K Scientific, #117042) (e.g., HYZ). Moreover, the TFA-to-HCl modification was taken for precaution, as the former is not an anion found naturally occurring in biological systems. Not unexpectedly, TFA is known to introduce confounding effects in both cellular and in vivo experiments (*61*, *62*). For experiments, both HYZ and HYZyne were prepared in parallel fresh from solid stocks in phosphate-buffered saline (PBS; pH 7.4) and adjusted to pH ~7 with 5 M NaOH the day of use. Both small molecules are soluble and stable under these conditions (50 to 100 mM).

#### 1-hydrazineyl-6-[(prop-2-yn-1-yloxy)methyl]phthalazine hydrochloride (e.g., HYZyne) purification

The crude product was purified on a C18 column by flash chromatography using H_2_O containing 0.1% HCl (A) and MeOH containing 0.1% HCl (B) as mobile phases with a 15-min gradient from 0 to 45% B. Fractions containing the product were combined, concentrated to remove organic solvents, and lyophilized to afford the desired product as a yellow solid (~95% yield): ^1^H nuclear magnetic resonance (600 MHz; MeOD) δ 8.83 (s; ^1^H), 8.38 (d; *J* = 8.5 Hz, ^1^H), 8.17 (s; ^1^H), 8.08 (dd; *J* = 8.6, 1.7 Hz, ^1^H), 4.91 (s; ^2^H), 4.37 (d; *J* = 2.4 Hz, ^2^H), and 2.98 (t; *J* = 2.4 Hz, ^1^H), as shown in fig. S22.

### Cell lines and culture conditions

All WT cells used in this study (HEK293T, MDA-MB-231, U-87, LN229, RAW 264.7, SH-SY5Y, and Neuro 2a) were purchased from American Type Culture Collection (ATCC). *ADO*-deficient SH-SY5Y cells were generated by CRISPR-Cas9–mediated gene editing as previously described (*18*). All cells were cultured in the manufacturer-recommended media supplemented with 10% (v/v) fetal bovine serum (FBS) at 37°C in a humidified 5% CO_2_ atmosphere. Specifically, HEK293T, MDA-MB-231, U-87, LN229, RAW 264.7, and Neuro 2a versus SH-SY5Y cells were expanded in high-glucose Dulbecco’s modified Eagle’s medium (DMEM; Gibco, #11995065) and DMEM/F12 (Thermo Fisher Scientific, #11320033) media, respectively. For alamarBlue assays, HEK293T, MDA-MB-231, U-87, and LN229 cells were passaged and analyzed in phenol red–free DMEM (R&D Systems, #M18650), which has the same composition as high-glucose DMEM with the exclusion of phenol red. For Stable Isotope Labeling by Amino acids in Cell culture (SILAC) experiments, each cell line was passaged a minimum of 6 times and maximum of 12 times in lysine- and arginine-free DMEM (Thermo Fisher Scientific, #A33822) containing dialyzed FBS (Silantes, #281001200) and isotopically enriched l*-*[^13^C_6_^15^N_2_]lysine hydrochloride and l*-*[^13^C_6_^15^N_4_]arginine hydrochloride (100 μg/ml each; Sigma-Aldrich) or natural abundance isotopologues (100 μg/ml each; 550 and 475 μM, respectively; Sigma-Aldrich) as described previously (*12*). All cells were passaged and maintained without antibiotics to preserve their integrity and ensure aseptic technique and tested on a biannual basis for mycoplasma contamination using MycoStrip (Invitrogen, #REP-MYS-20). All cells were treated, harvested, or otherwise evaluated in experiments within an eight-passage expansion lifetime to ensure integrity of the cells, their response to drugs, and reproducibility of the data.

### Animals for target identification in vivo

As we previously reported target profiling experiments and phenotypic characterization of knockout mice using the C57BL/6NJ strain (*15*), the same C57BL/6NJ mice (8 to 14 weeks and male; the Jackson Laboratory) were purchased and used for in vivo experiments in this study. All mice were housed with two to five mice per cage under a 12-hour normal light-dark cycle (light-on a.m., light-off p.m.) with ad libitum access to standard chow and water. Protocols (806885) were approved by the Institutional Animal Care and Use Committees at the University of Pennsylvania (Philadelphia, PA).

### In situ labeling of cells to identify HYZyne-reactive targets

Gel- and MS-based experiments to identify HYZ(yne)-reactive targets in cells were conducted essentially as previously described (*11*, *12*). At the time of treatment, cells were grown to 60 to 80% confluence, washed with cold PBS (pH 7.4), and replenished with serum-free DMEM (10% of normal passage volume) supplemented with 10 mM Na-Hepes buffer (pH 7.5). Unless otherwise indicated, cells were treated with HYZ(yne) (1 mM) for 0.5 hours at 37°C, either alone or following pretreatment with HYZ or iron chelators at 37°C. Concentrations and incubation conditions for HYZ and other inhibitors and/or chelators vary according to experiment type. Hence, they are included in Results and all figure legends. For MS-based experiments only, isotopically “light” cells were treated with HYZ (for 0.5 hours at the same concentration as HYZyne in “enrichment” experiments) or HYZyne following pretreatment (for 0.25 hours) in the presence of 10-fold HYZ (in “competition” experiments). Isotopically “heavy” cells were treated with HYZyne (for 0.5 hours) in both types of experiments. All treatments were performed in non-SILAC media. Exceptions include the following: (i) the gel-based HYZyne profiles evaluating endogenous (Fig. 1E) and overexpressed (Fig. 2, F and G) ADO in HEK293T soluble proteomes (HYZyne was 10 and 100 μM, respectively, instead of 1 mM); (ii) the MS-based HYZyne enrichment and competition profiles for U-87 cells (fig. S20) (HYZyne and HYZ were 100 and 500 μM, respectively, and appropriate for the corresponding experiment type). Following treatments, cells were harvested, and corresponding proteomes were fractionated as previously described (*11*, *12*).

### Gel-based analysis of HYZyne-labeled proteins

To visualize HYZyne-sensitive protein activity profiles, soluble and membrane cell or tissue proteomes were conjugated to rhodamine azide via copper-catalyzed azide-alkyne cycloaddition (CuAAC or “click” chemistry), resolved by SDS-PAGE, and visualized by in-gel fluorescence scanning as previously described (*11*), with the only exception being that shown in Fig. 1E. For this experiment only, the concentration of rhodamine azide used in the click reaction was reduced by 10-fold (2.5 μM). Here, the reduction in the overall fluorescence intensity of labeled protein bands could be compensated by a longer exposure time of the gel, thereby serving to minimize any background side chemistry from the click reaction that would be expected to be most apparent for highly abundant proteins in the lysate. These modified conditions served to enhance visualization of the HYZyne-reactive proteins in the molecular weight region for ADO (29 kDa).

### Identification of HYZyne-enriched targets from heart tissue

For target identification in heart proteome, C57BL/6NJ male mice were treated intraperitoneally with HYZ or HYZyne (12.5 mg/ml in PBS, pH adjusted to ~7 with 5 M NaOH) for 4 hours at 50 mg/kg, following proteome preparation and analysis protocols as previously described (*15*).

### MS-based quantitative analysis of HYZyne-labeled proteins from cells and tissues

Proteomes were similarly conjugated to biotin azide by CuAAC, and HYZyne-labeled proteins were enriched by streptavidin affinity chromatography and proteolytically digested on bead with trypsin as previously described (*11*, *12*). Specific to heart and brain mouse tissue proteomes (Figs. 1F and 5G described below, respectively), tryptic peptide digests separately processed from HYZyne- versus HYZ-treated animals were then differentiated using isotopologues of formaldehyde (CH_2_O) (^13^C- and ^2^H-enriched heavy versus natural abundance light, respectively) via late-stage reductive dimethylation (ReDiMe) of corresponding N termini and lysine residues (*20*). HYZyne-enriched peptides were identified and quantified by ratiometric comparison of heavy/light ReDiMe ratios, as described previously (*15*).

### Liquid chromatography–tandem MS analysis of tryptic digests

Peptide mixtures from tissue or cell proteomes were desalted before identification (based on MS2 spectra assignments) and quantification (based on intensity ratio of extracted MS1 parent ion chromatograms for corresponding coeluting isotopic peptide pairs) by liquid chromatography–tandem MS (LC-MS/MS)–based proteomics and analyzed according to previously reported methods (*11*, *63*, *64*). Selection criteria for a high-value target are as follows: First, proteomes analyzed following treatment of the HYZyne probe (defined by its alkyne handle for biochemical enrichment) must “enrich” for the protein of interest when compared against the native HYZ drug (which has no handle for enrichment). If sufficient enrichment is achieved from cells harvested from HYZyne-treated cells versus that of HYZ-treated cells [enrichment ratio (ER)], then the protein can then be evaluated for competition by pretreating cells with HYZ before treatment with HYZyne. If the HYZ drug blocks enrichment by the HYZyne probe [competition ratio (CR)], then this criterion indicates high reactivity of the target toward the drug and high occupancy of the target by the drug. This parameter is critical for determining small-molecule target engagement and stoichiometry and is useful in assessing the likelihood that engagement of a specific target may produce a therapeutic response (e.g., efficacy) or cause deleterious side effects in patients (e.g., safety). Proteins both substantially enriched by the HYZyne probe (ER > 5) and competed by the HYZ drug (CR > 3) were considered high-reactivity and high-stoichiometry targets. Unless otherwise noted below, “heavy/light” SILAC or ReDiMe protein ratios were derived from the median of three or more unique quantified peptides per protein and averaged across replicates (as explicitly defined in data S1 and S2) to generate the final ratios, as previously described (*15*). For mouse tissues, protein ER and CR values were determined using the median ratio from two or more unique peptides, while for U-87 cells, one or more unique peptides were used.

### Cloning and transfection of overexpression constructs in HEK293T cells

All genes were cloned into mammalian expression vectors (pRK5) containing *C*-terminal FLAG affinity tags (*11*). In this construct, the gene products are expressed with an additional 15 amino acids (A_3_G_4_DYKD_4_K) appended to their C terminus. The full-length human gene encoding *ADO* was amplified from a cDNA library derived from the same low-passage HEK293T cells used for proteomic profiling as reported previously (*11*). The coding sequence cloned from cells matched the coding sequence in GenBank (BC018660.1 and AK127694.1) that corresponded to full-length *ADO*. Incidentally, the coding sequence for the human *ADO* gene amplified from our HEK293T cells, which we directly purchased from ATCC at the time, happened to harbor two base pair mutations that corresponded to G25T and P39A variants when compared to the National Center for Biotechnology Information (NCBI)–assigned reference sequence for *ADO* (NM_032804.6). Ironically, this includes the same P39A substitution that is a missense variant commonly found in the population that is associated with decreased blood pressure (*21*, *22*) and discussed above. Active-site mutants of ADO were generated using QuikChange II XL site-directed mutagenesis (Agilent) using primers (Integrated DNA Technologies) containing the desired mutations and their respective complements. All constructs were verified by DNA sequencing. Human *RGS5* cDNA was purchased (GenScript, OHu22885) and subcloned into the same pRK5-flag vector above at *SalI* and *NotI* restriction sites.

ADO and RGS5 were transiently overexpressed in low-passage HEK293T cells grown to 40 to 60% confluency under standard conditions using polyethyleneimine “MAX” (molecular weight: 40,000; Polysciences Inc.) as previously described (*11*). Cells were incubated for ~48 hours before harvesting or labeling in situ. Proteomes were processed for gel- and MS-based experiments as described in other sections.

### Site- and structure-based resolution of HYZyne-captured peptides from ADO-overexpressing cells

To identify the basis for ADO inhibition by HYZyne and HYZ, we isolated and characterized the HYZyne-modified peptides from probe-treated HEK293T cells overexpressing recombinant human ADO (hADO) as adopted from previous studies (*11*, *23*). Briefly, the resultant HYZyne-labeled peptides are identified as coeluting isotopic pairs in a 1:1 ratio that are resolved by 6.0138 kDa due to incorporation of natural abundance (light) or l-[^13^C_5_^15^N]valine (heavy) into the portion of the tag that is retained, enabling all probe-labeled peptides regardless of their mass or identity to be distinguished from other ions in the sample by their unique pair feature with a defined mass differential.

### Purification and reconstitution of active hADO from *Escherichia coli*

The gene for *hADO* (NCBI reference sequence: NP_116193.2) was codon optimized and subcloned into pET-28a for use in recombinant expression and purification in BL21(DE3) *E. coli* as previously described (*25*, *26*). Briefly, bacterial cultures were grown at 37°C in M9 minimal medium and induced for 5 hours at 28°C via addition of 0.5 mM isopropyl-β-thiogalactoside supplemented with 20 μM CoCl_2_ or (NH_4_)_4_Fe(CN)_6_ for Co(II)•ADO or Fe(II)•ADO, respectively. Harvested cells were resuspended in lysis buffer [50 mM tris-HCl with 200 mM NaCl (pH 8.0)] for mechanical disruption with an LM20 Microfluidizer. Cell debris was removed by centrifugation (34,000*g* for 40 min) at 4°C, and ADO protein in the recovered supernatant was captured onto Co- or Ni–nitrilotriacetic acid agarose beads and eluted by the addition of imidazole (300 mM) to the same buffer. The crude His^6^-tagged ADO protein was then dialyzed in 10 mM tris-HCl with 5% glycerol (pH 8.0) overnight at 4°C in the presence of Tobacco Etch Virus (TEV) protease to cleave the His^6^ tag used for affinity purification. Cleaved hADO protein was further purified using an in-house packed Superdex 75 (Cytiva) gel-filtration column (16/600, 120 ml), equilibrated with 50 mM tris-HCl and 50 mM NaCl (pH 7.6). After purity assessment by SDS-PAGE (~98%), the proteins were either concentrated for subsequent experiments or stored in 50 mM tris-HCl, 50 mM NaCl, and 5% glycerol (pH 7.6) and flash frozen at −80°C. Protein concentration was determined on the basis of its calculated extinction coefficient of ε_280nm_ = 22,920 cm^−1^ M^−1^ (*65*) (https://web.expasy.org/protparam/). As in prior studies, the WT enzyme was used for kinetic and spectroscopic assays, whereas the C18S/C239S variant was used for crystallography, as previously described.

### Structural characterization of Co(II) versus Fe(II)•ADO with HYZ by x-ray crystallography

The crystallization was achieved using the hanging drop vapor-diffusion method at 289 K with a buffer containing 0.1 M Bis-tris (pH 5.5), 0.2 M (NH_4_)_2_SO_4_, and 20% (w/v) polyethylene glycol, molecular weight 3350. Microcrystals formed after 1 to 2 days and reached an optimal size for x-ray diffraction after 7 days. The Co(II)•ADO or Fe(II)•ADO crystals (*26*) were soaked with HYZ (50 mM for 0.5 hours) and cryoprotected with crystallization buffer containing an additional 25% (v/v) glycerol before being flash cooled in liquid N_2_. Crystallographic data were collected at the Stanford Synchrotron Radiation Lightsource beamline BL 9-2 at 100 K. All x-ray diffraction intensity data were integrated, scaled, and merged using HKL-3000 (*66*). Molecular replacement was performed with Phenix (*67*) using the structure of Ni-hADO [Protein Data Bank (PDB) entry: 7REI] (*25*) as an alignment model, which was manually adjusted and further refined using Coot (*68*) and Phenix (*67*). Ramachandran statistics were analyzed using MolProbity (http://molprobity.biochem.duke.edu/). All the molecular model figures were generated using PyMOL (W. L. DeLano, The PyMOL Molecular Graphics System version 1.8.6.0; Schrödinger LLC; https://www.pymol.org/; 2002). The structural data were deposited to the PDB database with entry codes 9DMA and 9DY4.

### Characterization of HYZ binding to Co(II)•ADO by EPR spectroscopy

Anaerobically prepared stock solutions (100 mM) of HYZ, cysteamine, or buffer control [50 mM tris-HCl (pH 7.6)] were added to a concentrated stock of Co(II)•ADO under anaerobic conditions. The three samples were transferred to quartz EPR tubes and slowly frozen in liquid N_2_. EPR spectra were recorded as described previously (*26*) on a Bruker E560 X-band spectrometer equipped with a cryogen-free 4-K temperature system with an SHQE high-Q resonator, operating at a modulation frequency of 100 kHz, with 0.6-mT modulation amplitude, and averaging four scans per spectrum. Full-range scans were conducted at 3.17-mW microwave power at 30 K.

### Inhibition of Fe(II)•ADO enzyme activity by HYZ

Iron loading of the enzymes was quantified using the ferrozine assay (*69*). Inhibition assays were conducted using an oxygen electrode (Oxygraph, Hansatech Instruments) as previously described (*25*) and shown in fig. S11 (A and B). Briefly, enzyme (2 μM) was mixed with excess ascorbic acid (20 μM) and HYZ (0, 6.7, or 10 μM) and stirred in the chamber for 2 to 3 min until mixed and the signal for O_2_ consumption stabilized (defined as “premix phase” in fig. S11B). To initiate catalysis, varying concentrations of cysteamine (0 to 48 mM) were injected into the chamber solution through a 10-μl syringe (defined as “injection point”). O_2_ consumption was monitored continuously for ~5 min while maintaining a constant stirring speed of 100 rpm. A 1-min interval following injection was selected to determine the initial rates (defined as “initial phase”), and the background rates in the absence of cysteamine were subtracted from the initial rates. The reaction mixture (total volume: 0.5 ml) consisted of 100 mM tris-borate buffer (pH 8.0; 25°C). Net oxygen consumption was determined by subtracting the initial oxygen consumption rate in the absence of the enzyme from that in the presence of the enzyme. All experiments were performed in triplicate. The three lines in fig. S11A were generated by plotting the reciprocal of net oxygen consumption against the reciprocal of enzyme concentration at different HYZ concentrations. Global fittings were conducted across the datasets using OriginPro (OriginLab), where the parameters *k*_cat_, *K*_m_, *K*_i1_, and *K*_i2_ are shared in the following equation

$$\frac{1}{V_{0}}=\left[\left(1+\frac{\left[/\right]}{K_{i1}}\right)\frac{K_{m}}{k_{\text{cat}}}\frac{1}{\left[S\right]}\right]+\left(1+\frac{\left[/\right]}{K_{i2}}\right)\frac{1}{k_{\text{cat}}}$$

Here, the equation includes two *K*_i_ values. *K*_i1_ reflects the dissociation rate constant for competitive inhibition (where HYZ binds directly to the iron center of ADO; e.g., at the substrate binding site), whereas *K*_i2_ reflects that for uncompetitive inhibition (where HYZ binds to an allosteric site that does not compete with that for primary substrate). In other words, the data points to a mixed inhibition pattern where HYZ may bind to both the substrate-free and substrate-bound complexes with different affinities. This is consistent with both the crystallography and spectroscopy data that showed that HYZ binding can occur not only to the Fe center but also potentially at a peripheral site.

### Protein expression analysis by Western blotting

HEK293T and SH-SY5Y cells were harvested for protein expression as previously described (*11*, *12*) with the following modifications. Cells were harvested in radioimmunoprecipitation assay buffer [e.g., 300 μl of lysis buffer; 25 mM tris-HCl (pH 7.6), 150 mM NaCl, 1% NP-40, 1% sodium deoxycholate, and 0.1% SDS; Thermo Fisher Scientific, #89900] and further sonicated to shear genomic DNA (10 pulses; 0.3- and 2-s on and off, respectively; and 15% duty cycle). Total protein concentration was determined by the DC assay (Bio-Rad) on a microplate reader (*11*, *12*). Protein samples (20 μg) were resolved by SDS-PAGE (Novex tris-glycine; 16% acrylamide was ideal for ADO and RGSs, which are all <30 kDa) and transferred to polyvinylidene difluoride membrane in Towbin buffer, and the membrane was blocked for ~1 hour at ambient temperature with 5% nonfat dry milk (w/v) in tris-buffered saline with 0.05% Tween 20 (TBST) and incubated with primary antibodies in the same solution overnight at 4°C or 1 hour at room temperature as reported previously (*12*). Specific to this study, the primary antibodies included the following: anti-FLAG (1:2500; F1804, Sigma-Aldrich), anti-ADO (1:1000; ab134102, Abcam), anti-RGS4 (1:250; D4V1P, Cell Signaling Technology), anti-RGS5 (1:100; sc-514184, Santa Cruz), anti-RGS16 (1:100; sc-166083, Santa Cruz), anti–HIF-1α (1:1000; 610959, BD Biosciences), anti–glyceraldehyde-3-phosphate dehydrogenase (GAPDH; 1:1000; MA515738, Invitrogen), and anti–β-actin horseradish peroxidase (HRP; 1:10,000; ab49900, Abcam). Blots were washed (3 × 5 min; TBST), incubated with the appropriate secondary antibodies listed below in 5% milk for 1 hour at room temperature, washed again (3 × 5 min; TBST), and visualized on a ChemiDoc MP Imaging System (Bio-Rad). For fluorescent detection, goat anti-rabbit Alexa Fluor 488 (1:2000; A-11034, Invitrogen) was used as a secondary antibody. For chemiluminescent detection, goat anti-mouse HRP (1:10,000; ab150113, Abcam), goat anti-rabbit HRP (1:2000; 65-6120, Invitrogen), or sheep anti-mouse HRP (1:2000; NA931V, Sigma-Aldrich) coupled with the HRP substrate, SuperSignal West Atto (Thermo Fisher Scientific, #A38554). Specific for detection of ERK1/2 and phospho-ERK1/2, 5% milk was replaced with 3% bovine serum albumin (w/v) for both primary and secondary antibody solutions, as necessary to avoid cross-reactivity with abundant phosphoproteins (e.g., casein) known to be present in the blocking milk. Antibodies used for this analysis were anti-ERK1/2 (1:2000; 9102, Cell Signaling Technology) and anti–phospho-ERK1/2 (1:2000; 9101, Cell Signaling Technology).

### Determination of IC_50_ values

We used competitive ABPP (*13*, *16*, *70*, *71*) to determine in situ IC_50_ values for inhibitor molecules that block the active site and prevent HYZyne probe attachment by quantifying the diminution in band intensity as a function of increasing drug or inhibitor concentration using ImageJ (Figs. 1D and 2G and fig. S7). Data were fitted to a dose-response curve to determine IC_50_ values by GraphPad Prism 10 using nonlinear regression analysis as described previously (*13*). Similarly, to determine the IC_50_ for HYZ required to stabilize levels of protein substrates via ADO inhibition, substrate levels were quantified by Western blot with the same analysis (Fig. 4B and fig. S13).

Specifically, to assess the selectivity of HYZ for ADO and against PHD enzymes, their substrate levels (RGS5 and HIF-1α, respectively) were quantified simultaneously by each band intensity measured by Western blot (fig. S15). Here, substrate protein levels were normalized according to the corresponding band intensity for β-actin, which served as a loading control. The average (*n* = 3) maximum substrate/β-actin ratio was scaled to 100% and plotted as a function of [HYZ]. Data were fitted to a dose-response curve to determine IC_50_ values by GraphPad Prism 10 using nonlinear regression analysis as described previously (*13*).

### FerroOrange assay of intracellular Fe(II)

As described previously (*27*), SH-SY5Y cells were seeded in black, clear-bottomed 96-well plates and allowed to reach 80% confluency before being treated with the indicated concentrations of Dp44mT or HYZ for 20 hours in low-autofluorescence DMEM (Thermo Fisher Scientific, #A1896701) containing 1% FBS. The medium was then replaced with imaging buffer (117 mM NaCl, 4.5 mM KCl, 25 mM NaHCO_3_, 11 mM glucose, 1 mM MgCl_2_, 1 mM NaH_2_PO_4_, and 1 mM CaCl_2_) containing 1 μM FerroOrange (Sigma-Aldrich, #SCT210). Cells were incubated for 0.5 hours at 37°C, and then the intensity of fluorescence emitted at 590 nm, when excited at 544 nm, was measured using a plate reader (Spark, Tecan). Fluorescence from a well containing medium alone (e.g., no FerroOrange) was used as a blank, and data are reported as the percentage of change in fluorescence relative to untreated control cells (e.g., in the absence of Dp44mT and HYZ) (Fig. 4D).

### Cell-based GAP assay

To evaluate ADO- and HYZ-dependent changes in the deactivation kinetics of Gα_q_, a critical upstream regulator of Ca^2+^ signaling directly modulated by RGSs, we measured the impact of HYZ on the GAP activity of RGS proteins using a BRET assay, as previously described (*37*). This system allows for highly sensitive, real-time monitoring of signaling events across major G protein families upon activation of GPCR (e.g., M_3_R) (e.g., by the neurotransmitter acetylcholine, an M_3_R agonist). For this study, subsequent binding of an M_3_R antagonist (e.g., atropine) then triggers deactivation of the same receptor, the rate by which is sensitively regulated and enhanced by the intrinsic GAP activity of RGS proteins.

High-affinity ligand binding of an agonist at an extracellular-facing receptor site activates G proteins (α, β, and γ) that are bound to an intracellular-facing site, which occurs by ligand-triggered release of Gα-bound guanosine diphosphate (inactive state) in exchange for its energetic equivalent, guanosine 5′-triphosphate (GTP). Coupled to dissociation of the Gβγ dimer from GTP-bound Gα, a rapid and targeted signal transduction pathway is initiated on the basis of information directly transmitted by the ligand through the receptor. The BRET system used here allows for specific detection of dissociation of Gα from Gβγ upon GPCR activation (Fig. 4E), as described above and previously in extensive detail (*37*, *72*). The resultant Gβγ harbors a Venus-based fluorescence acceptor that specifically recognizes a fragment protein G protein–coupled receptor kinase 3 (GRK3; masGRK3ct) containing a “NanoLuc” fluorescent donor to pair. Hence, the interaction between Gβγ-Venus and masGRK3ct-NanoLuc generates a BRET signal proportional to G protein activation. In this study, we pretreated this robustly engineered system with acetylcholine, an M_3_R agonist, to fully activate G protein signaling, followed by atropine, an M_3_R antagonist, to initiate rate measurements for G protein deactivation as a function of cellular concentrations of RGS protein expression. To directly measure the change in *k*_GAP_ as a function of ADO activity and consequent inhibition by HYZ in situ, *k*_GAP_ was measured in the absence and presence of *ADO* expression plasmids and HYZ (Fig. 4, E and F, and figs. S16 to S18). To establish the expression system, HEK293T/17 cells (2 × 10^6^ per well) were transfected with the following constructs for 4 hours: M_3_R (0.21 μg), Venus 156-239-Gβ_1_ (0.21 μg), Venus 1-155- Gγ_2_ (0.21 μg), masGRK3ct-Nluc-HA (0.21 μg), Gα_q_ (0.42 μg), RGS4 (2.52 μg), RGS5 (2.52 μg), RGS16 (0.63 μg), and ADO (1.22 μg). pcDNA3.1+ was used to normalize the amount to 5 μg for each transfection. As previously described (*37*), cells (5 × 10^4^ per well) were replated in 96-well flatbottomed white microplates (Greiner Bio-One) and treated, as specific to this study, with buffer or various concentrations of HYZ for 1 hour. Deactivation kinetics of Gα were recorded as previously described (*37*). The rate constants (1/τ) of the deactivation phases were obtained by fitting the one-phase decay model to the traces using GraphPad Prism 10.

### Intracellular calcium measurement

SH-SY5Y neuroblastoma cells (7 × 10^4^) were seeded in 96-well plates (Sigma-Aldrich, #CLS3603) and cultured for 24 hours before cotreatment with varying concentrations of HYZ (0 to 100 μM for 1 hour) in the presence of the ratiometric and ultraviolet light-excitable intracellular Ca^2+^ indicator, Fura-2 AM, as prepared in assay buffer and used according to the manufacturer’s instructions (Abcam, #ab176766). The change in intracellular Ca^2+^ (ΔCa^2+^; Fig. 4G) as a function of GPCR-dependent (e.g., M_3_R) Ca^2+^ signaling triggered by the agonist, carbachol, and its attenuation in response to HYZ as a function of direct control of RGS levels was determined using the ratio of Fura-2 AM fluorescence at 510 nm when excited at 340 and 380 nm (*F*_340_/*F*_380_). Measurements were performed at 21-s intervals at 37°C using an Infinite M1000 plate reader (Tecan) up to 532 s, carbachol (M_3_R agonist; 100 μM) was added to induce GPCR-dependent Ca^2+^ release, and acquisition was reinitiated at 128 s. To determine the ΔCa^2+^, the minimum *F*_340_/*F*_380_ ratio (*F*_min_) recorded during the basal period (between 0 and 128 s) was subtracted from the maximum *F*_340_/*F*_380_ ratio (*F*_max_) observed following the addition of carbachol (between 149 and 532 s).

### Effects of HYZ on cell growth and proliferation

1) Three-day assays: U-87, LN229, HEK293T, and MDA-MB-231 cell lines (1500 cells for 96-well plates or 2 × 10^5^ cells for 6-cm dishes) were seeded and cultured for 24 hours before treatment with HYZ (0.1 to 100 μM) versus buffer control (e.g., PBS) for 3 days. Cell growth was quantified for each condition using the alamarBlue assay, as described below (Fig. 5C). For U-87 cells only, crystal violet staining (Fig. 5D) and quantitative polymerase chain reaction (qPCR; Fig. 5F), as described below, were used to assess morphology, cytotoxicity of HYZ, and gene expression, respectively.

2) Nine-day time course: U-87 cells (500 cells for 96-well plates) were seeded and incubated for 24 hours before treatment with HYZ (at 10 or 100 μM) versus buffer control (e.g., PBS). Each day for 9 days, growth was quantified using the same alamarBlue assay as above (Fig. 5B).

3) Twelve-day (long-term) time course: U-87 cells (250 for 96-well plates or 1 × 10^4^ for 6-cm dishes) were treated in the same manner as above with the exception that single treatment was compared to repeated treatment (3-day intervals). Cell growth and morphologies were measured by the alamarBlue assay and crystal violet staining, respectively (Fig. 5E).

#### alamarBlue assay

Cell viability was quantified on the basis of reduced nicotinamide adenine dinucleotide (NADH) levels in live cells (*73*). Cells were treated by addition of alamarBlue HS (3 hours) (Invitrogen, #A50100). The number of live cells in each well was quantified on the basis of the intensity of fluorescence at 590 nm when excited at 560 nm, as measured on an Infinite M1000 (Tecan) fluorescence plate reader. Background fluorescence from cell-free medium in the presence of alamarBlue HS was subtracted from sample fluorescence reported.

#### Crystal violet staining

Cell density and morphology were evaluated with this pan-DNA and protein stain (Sigma-Aldrich, #C6158). Cultured cells were fixed with 4% paraformaldehyde for 10 min and stained with crystal violet solution [0.5%; methanol:water = 20:80 (v/v)] for 0.5 hours at room temperature. Cells were then washed to remove residual crystal violet. The image showing cell morphology was acquired by an Eclipse TE2000-U (Nikon) microscope. The image of cell density was acquired using an iPhone 15 Pro.

#### Quantitative PCR

mRNA expression levels were measured using real-time quantitative reverse transcription (qRT)–PCR. Total RNA was purified from cells with the RNeasy Mini Kit and deoxyribonuclease I (Qiagen, #74104 and 79254). cDNA was synthesized from 1 μg of mRNA with iScript Reverse Transcriptase (Bio-Rad, #1708841). qPCR was performed with QuantStudio 6 Flex Real-Time PCR System (Thermo Fisher Scientific) and Luna Universal qPCR Master Mix (New England Biolabs, #M3003). Cycle threshold (*C*_t_) values for senescence marker expression in U-87 cells were normalized to *C*_t_ values of 18*S* ribosomal RNA. Relative expression levels of each marker were calculated using the ΔΔ*C*_t_ method (*74*) and normalized against PBS controls. Markers with undetermined *C*_t_ values due to high cycle numbers were classified as “not detected” in the figure. Primer sequences were based on previous literature (*75*) and provided in data S3.

### Measurement of cell death

Cytotoxicity was evaluated by counting the number of dead cells using double staining with live-cell–permeable and –impermeable DNA dyes (fig. S21), as previously described (*76*). U-87 cells (2 × 10^5^ cells for 6-cm dishes) were seeded and cultured for 24 hours before treatment with HYZ (0.1 to 100 μM) versus buffer control (e.g., PBS). After 6 or 24 hours, cells were stained with Hoechst 33342 (10 μg/ml) (BD Pharmingen, #BDB561908) and propidium iodide (1 μg/ml) (BD Pharmingen, #BDB556463) for 0.5 hours at 37°C. Positive control cells were simultaneously treated with 0.1% Triton X-100. Images were acquired on an Eclipse TE2000-U (Nikon) microscope.

### Target identification in the brain by intracerebroventricular delivery of HYZyne versus HYZ

For the intracerebroventricular brain delivery experiment, immunodeficient NSG (NOD scid gamma mouse) mice (6 to 14 weeks) were obtained from the Stem Cell and Xenograft Core at the University of Pennsylvania. As described previously (*77*), anesthesia was induced with inhaled isoflurane (1 to 4%) and confirmed via pedal reflex before incision. The surgical area was prepared by shaving the head (Wahl Arco Cordless Clipper) and sterilizing the skin with alcohol prep pads. A midline incision was made using a #10 scalpel. A digital stereotaxic frame (Stoelting, #51730D) and a hand drill (Stoelting, #51449) were used to create burr holes in the skull. The bregma was identified, and a 10-μl Hamilton syringe (Hamilton, #80301) was positioned 1 mm lateral to the left and 0.3 mm anterior to bregma. The syringe was lowered until it touched the brain surface, zeroed, and advanced 3 mm deep. HYZyne or HYZ was injected in 1.5 μl (30 μg dissolved in 0.9% saline, adjusted to pH ~7 with 5 M NaOH). The incision was closed with skin glue (3M Animal Health, #1469SB), and mice were allowed to recover. After 4 hours, mice were euthanized; brain tissues were harvested; and proteomes were processed, ratiometrically compared, and analyzed as described previously and above for heart tissue (*15*).

## Supplementary Materials

### The PDF file includes:

Figs. S1 to S22 Tables S1 to S3 Legends for data S1 to S3 References
