# Supplementary material for: Hydralazine inhibits cysteamine dioxygenase to treat preeclampsia and senesce glioblastoma
Source: Sci Adv. 2025 Oct 15;11(42):eadx7687. doi: 10.1126/sciadv.adx7687 (PMC12526720; doi:10.1126/sciadv.adx7687)
Supplement: Supplementary file 1 — Figs. S1 to S22 Tables S1 to S3 Legends for data S1 to S3 References [file sciadv.adx7687_sm.pdf]

Science Advances
AAAS

Supplementary Materials for

Hydralazine inhibits cysteamine dioxygenase to treat preeclampsia and senesce glioblastoma

Kyosuke Shishikura *et al.*

Corresponding author: Kyosuke Shishikura, kyosuke@sas.upenn.edu;
Megan L. Matthews, megamatt@sas.upenn.edu

Sci. Adv. **11**, eadx7687 (2025)
DOI: 10.1126/sciadv.adx7687

The PDF file includes:

Figs. S1 to S22
Tables S1 to S3
Legends for data S1 to S3
References

Other Supplementary Material for this manuscript includes the following:

Data S1 to S3

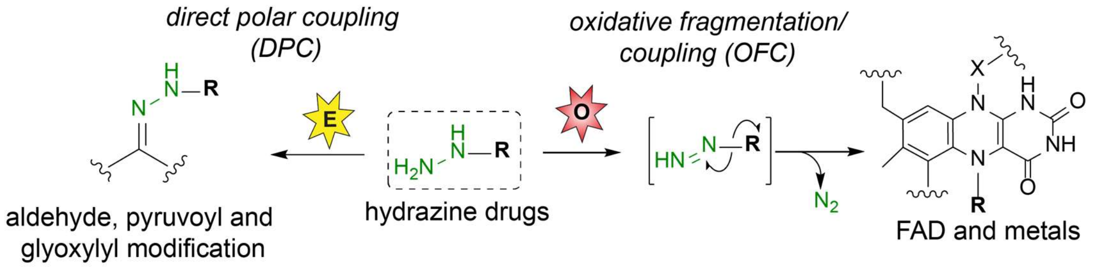

Fig. S1. Modes of hydrazine reactivity. Schematic for two distinct modes of probe reactivity: direct polar coupling (DPC) and oxidative fragmentation/coupling (OFC). Green depicts the reactive probe moiety.

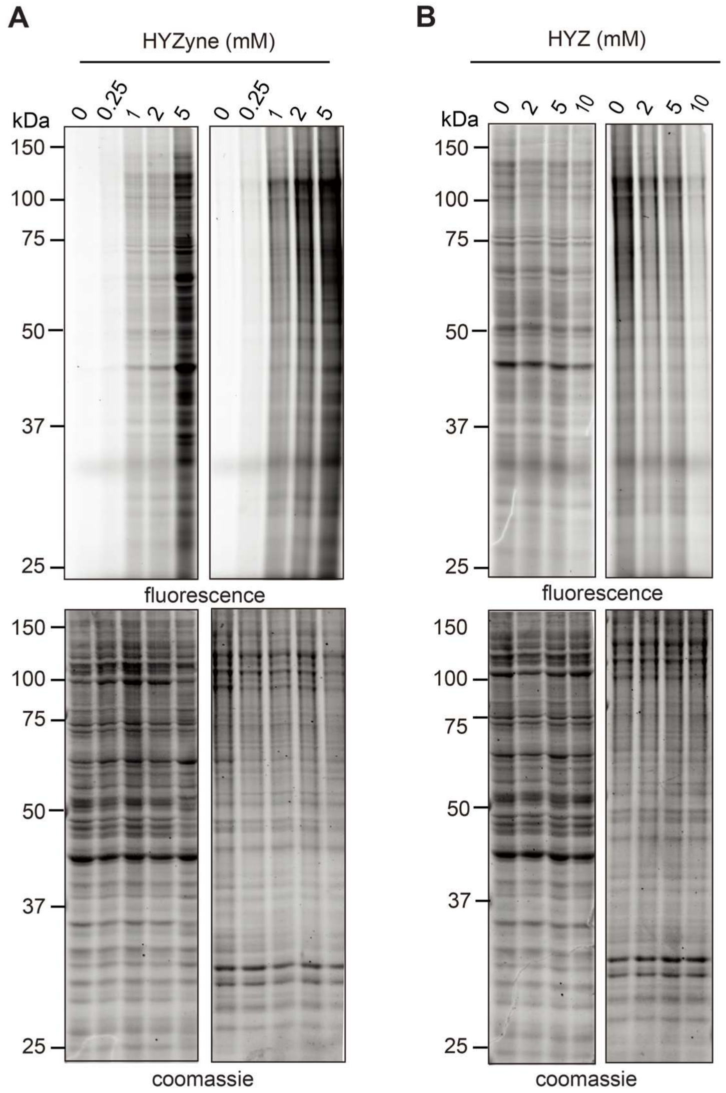

Fig. S2. Gel-based profiles for HYZyne-treated HEK293T cells. (**A**) Concentration-dependent labelling profiles (upper) for soluble (left) and membrane (right) proteomes of HYZyne-treated HEK293T cells (0.5 h). Corresponding expression profiles are shown (lower). (**B**) Competition of HYZyne labelling by HYZ (upper) for soluble (left) and membrane proteomes (right) of HEK293T cells pretreated with varying concentrations of HYZ (0.25 h; 2-10 mM) followed by 1 mM HYZyne treatment. Corresponding expression profiles are shown (lower).

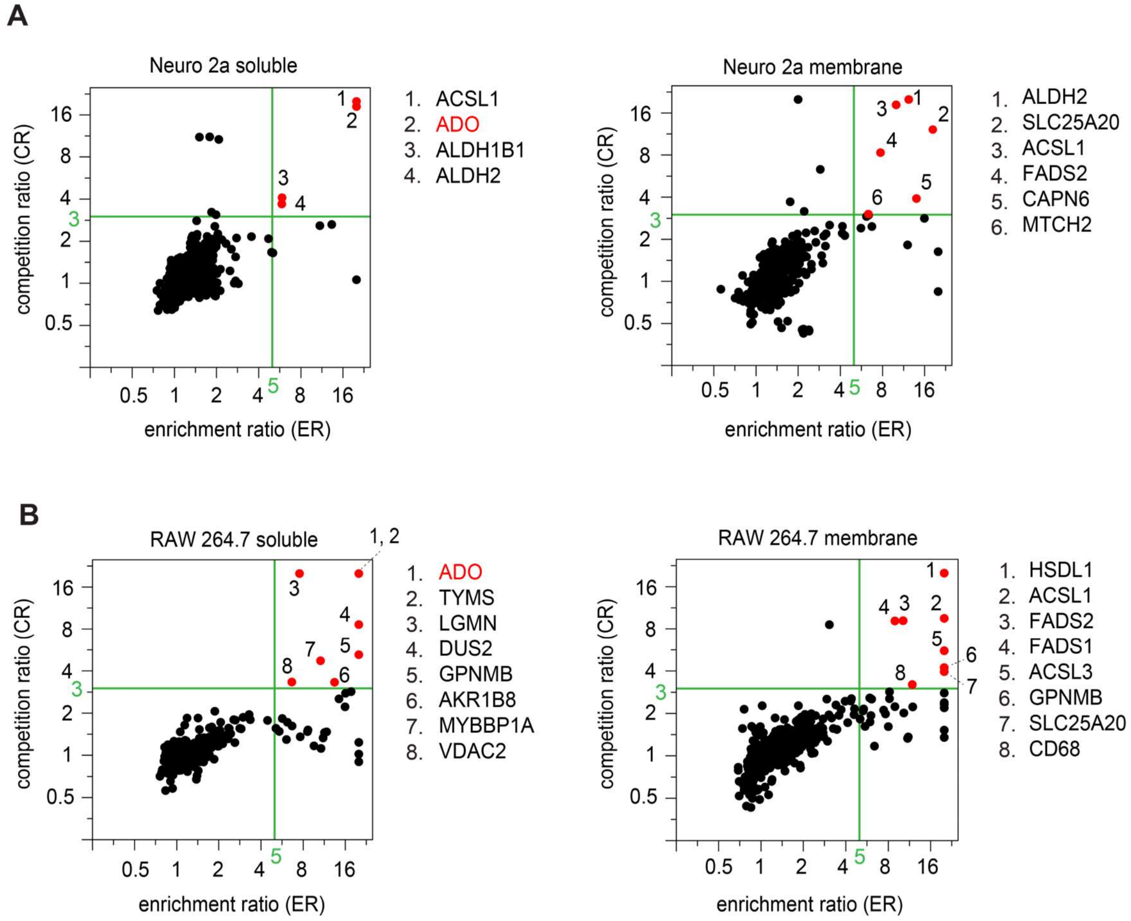

Fig. S3. Identification of protein targets of HYZyne in two mouse cell lines. (**A, B**) Quadrant plot of average enrichment [HYZyne (heavy cells) vs. HYZ (light cells); both treated with 1 mM for 0.5 h] versus competition [HYZyne (heavy cells) vs. HYZyne (light cells) where light cells were first pretreated with 10 × HYZ (10 mM; 0.25 h) prior addition of HYZyne (1 mM)]. SILAC ratios from quantitative proteomics experiments for the soluble (left) and membrane (right) proteomes of Neuro 2a (**A**) or RAW264.7 (**B**) cells. Proteins with ER ≥ 5 and CR ≥ 3 (upper right quadrant) were considered high-occupancy targets; listed to the right of the plot.

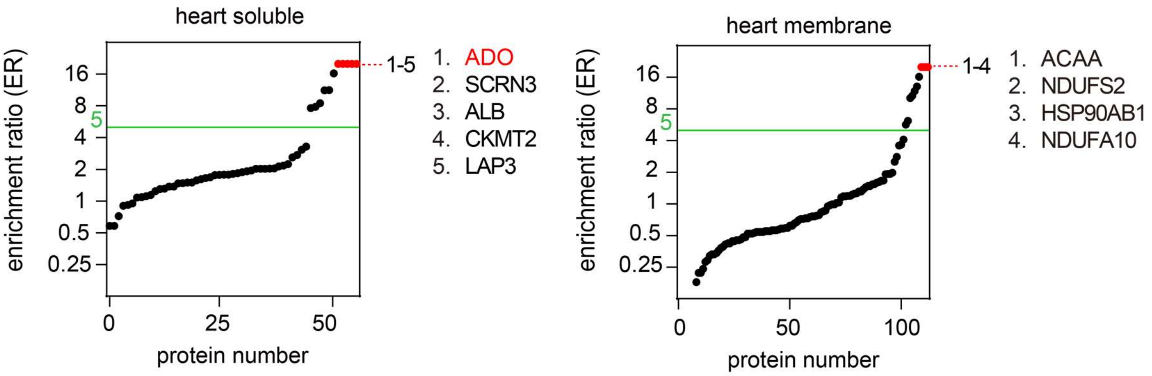

Fig. S4. High-reactivity targets of HYZyne in mouse heart. Average enrichment ReDiMe protein ratios versus protein number for HYZyne from quantitative proteomics experiments in the soluble (left) and membrane (right) proteomes of mouse heart 4 h post-injection via ratiometric comparison of HYZ (light) versus HYZyne (heavy) (50 mg/kg, intraperitoneally). Protein targets with ER ≥ 5 were considered high-reactivity targets. Proteins with ER ≥ 20 are annotated and are listed on the right of the plot.

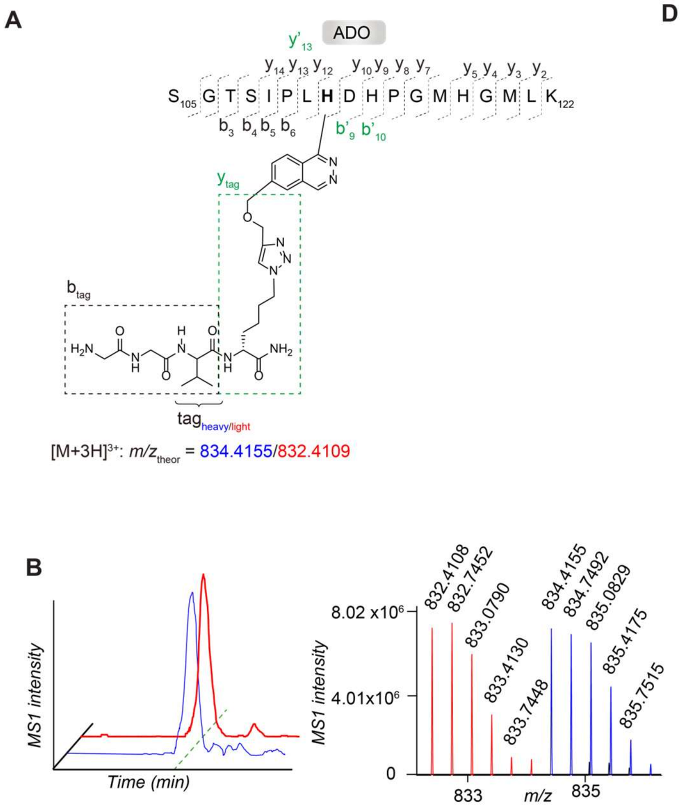

D

	Theoretical *m/z*		Measured *m/z*	
	Heavy	Light	Heavy	Light
b_{tag}	220.1330	214.1192	220.1332	214.1188
$[c_{13}-1]^{2+}$	967.4777	964.4708	967.4709	-
$[c_{17}-1]^{2+}$	1186.5802	1183.5733	1186.5763	-
y_{13}^{2+}	1029.0150	1026.0081	1029.0092	1026.0079
$[y_{13}-H_2O]^{2+}$	1020.0097	1017.0028	1019.9988	1016.9901
$[y_{13}-NH_3]^{2+}$	1020.5017	1017.4948	1020.5014	1017.4931
y_{12}^{2+}	980.4886	977.4817	-	977.4818
$[y_{12}-H_2O]^{2+}$	971.4833	968.4764	971.4791	968.4714
$[y_{14}-NH_3]^{3+}$	718.3649	716.3603	-	716.3618
a_3	218.1135		-	218.1133
a_5	418.2296		418.2299	418.2303
b_3	246.1084		246.1086	246.1086
b_3-H_2O	228.0979		-	228.0980
b_4	333.1405		333.1407	333.1405
b_4-H_2O	315.1299		315.1299	315.1302
$b_4-[H_2O]_2$	297.1196		297.1194	297.1196
b_5	446.2245		446.2246	446.2239
b_5-H_2O	428.2140		428.2141	428.2147
b_6-H_2O	525.2667		-	525.2672
b'_9	1275.6229		1275.6292	1275.6240
b'_{10}	1412.6818		1412.6884	1412.6901
y'_{13}	1836.8897		1836.8807	1836.8845
y_{10}	1122.5183		1122.5189	1122.5204
$y_{10}-H_2O$	1104.5077		-	1104.5116
y_9	1007.4914		1007.4906	1007.4922
y_8	870.4324		870.4326	870.4330
y_7	773.3797		773.3797	773.3790
y_5	585.3177		585.3179	585.3179
y_4	448.2588		448.2591	448.2590
y_3	391.2374		391.2366	391.2362
y_2	260.1969		260.1969	260.1971
MH'^{2+}	1141.5571		1141.5586	1141.5574
$y'_{13}{}^{2+}$	918.9485		918.9494	918.9517
y_8^{2+}	435.7199		435.7203	435.7186

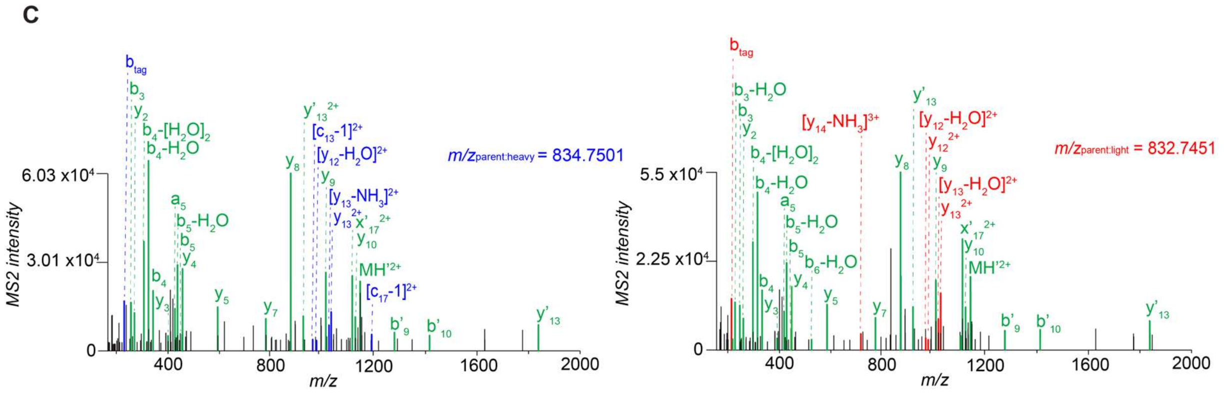

Fig. S5. MS/MS characterization of HYZyne-labelled active-site peptide of ADO. (**A**) Structures and theoretical parent masses of heavy- and light-tagged ADO peptides labelled by HYZyne and processed by the isoTOP-ABPP method. (**B**) **E**xtracted parent ion chromatograms and corresponding isotopic envelopes for heavy- (blue) and light- (red) tagged peptides quantified in human ADO-transfected HEK293T cells. (**C**) MS2 spectra generated from indicated parent ions (left: heavy and right: light). Unshifted ions are shown in green whereas fragment ions that retain the heavy or light portions of the tag are shown in blue and red, respectively. (**D**) Summary table of theoretical versus observed spectra assignments generated under high-resolution MS2 conditions.

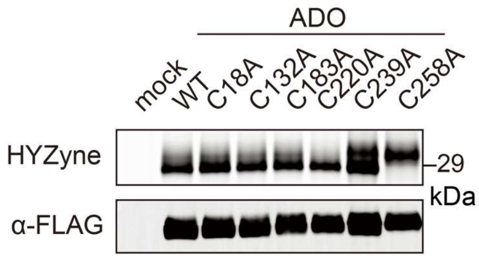

Fig. S6. HYZyne labelling of Cys mutants of ADO. HYZyne labeling of wild-type ADO and Cys-to-Ala mutant ADO proteins. Probe labeling (upper) and expression profiles (lower) for HYZyne-treated cells (1 mM, 0.5 h) overexpressing the indicated protein target or Cys mutants thereof.

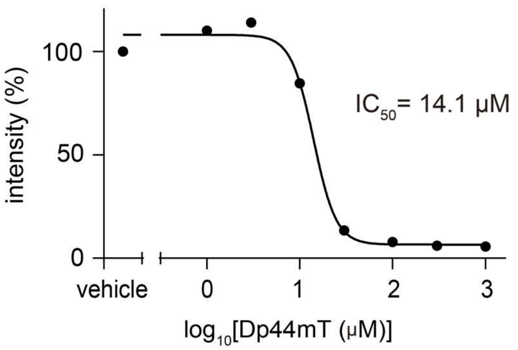

Fig. S7. Gel-ABPP-based inhibition curve of ADO by Dp44mT. IC_{50} values were determined by measuring HYZyne labeling (100 μM, 0.5 h) in the presence of increasing concentrations of Dp44mT as shown in Fig. 2G.

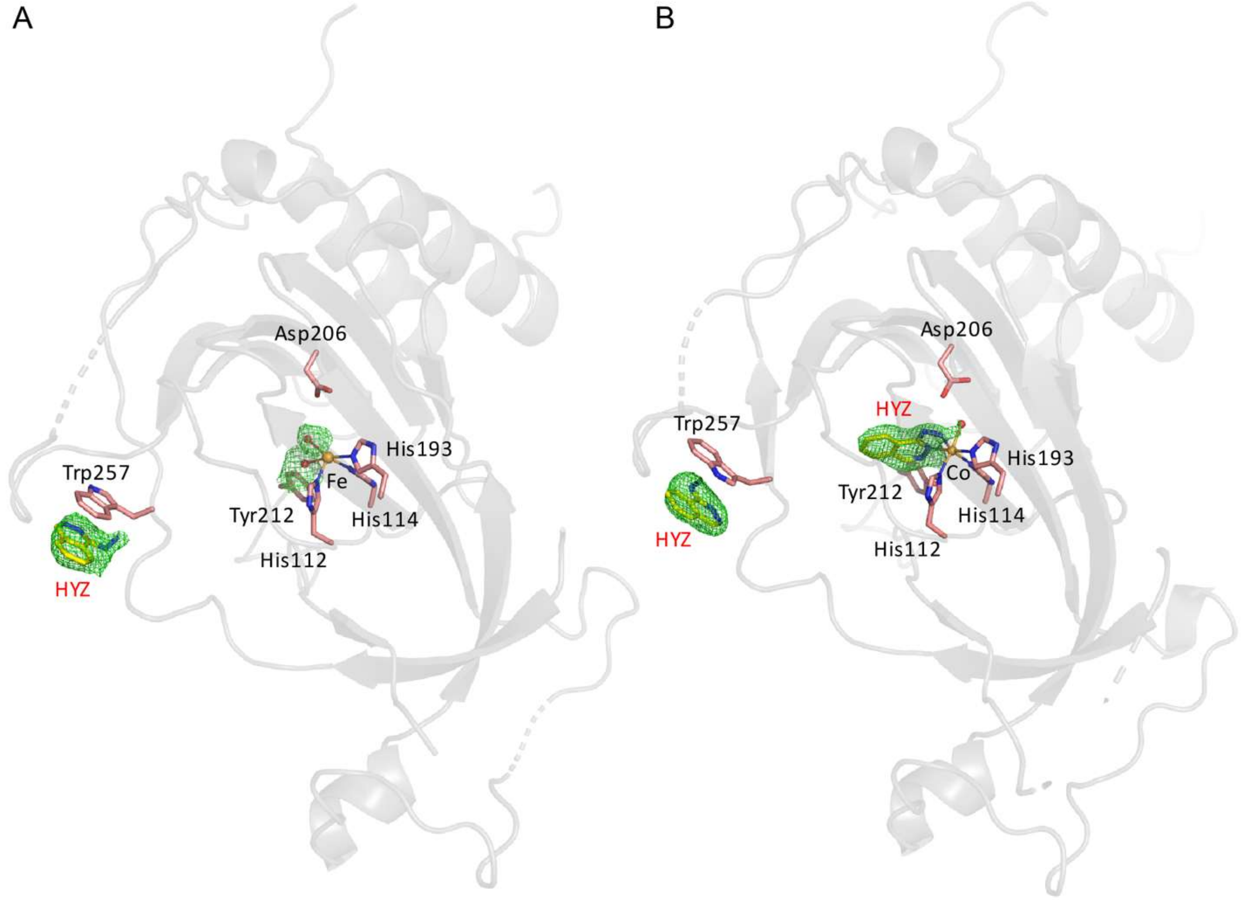

Fig. S8. X-ray crystal structures of HYZ-bound Fe(II)•ADO (A) and HYZ-bound Co(II)•ADO (B). The structure of HYZ-bound Fe(II)•ADO was determined at 2.39 Å resolution (PDB: 9DY4), and that of HYZ-bound Co(II)•ADO determined at 1.88 Å resolution (PDB: 9DMA). The *Fo–Fc* maps are contoured at 3.0 σ and shown in green.

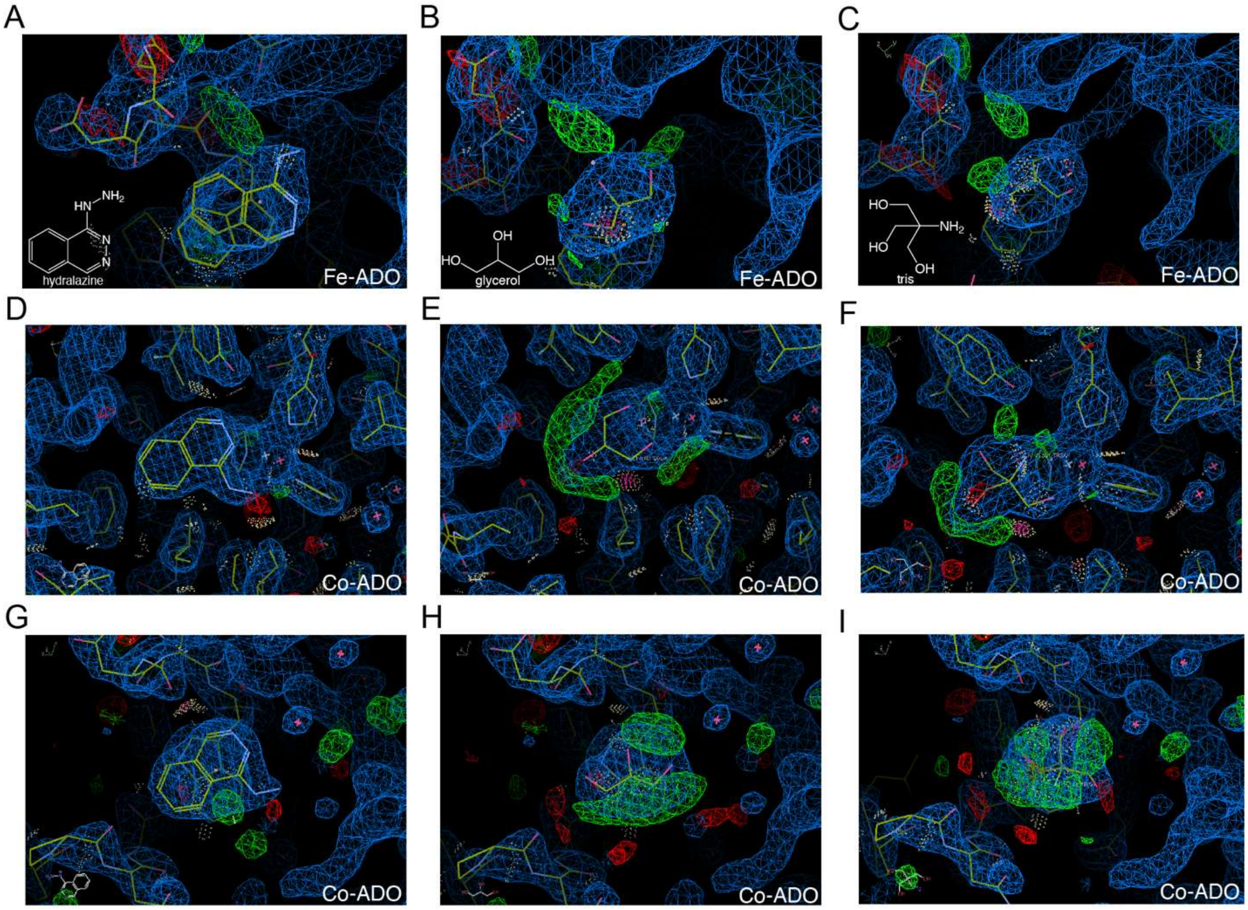

Fig. S9. Electron density maps for small molecules from the crystallization mother liquor in HYZ-soaked ADO crystals. Fe(II)•ADO and Co(II)•ADO crystals were soaked with 50 mM HYZ for 30 min, cryoprotected with crystallization buffer containing an additional 25% (v/v) glycerol before being flash-cooled in liquid N_2. 2*F*o–*F*c maps (blue) are contoured at 1.2 σ (**A-C**) or 1.5 σ (**D-I**). *F*o–*F*c maps (green) are contoured at 3.0 σ (**A-I**). (**A-C**) HYZ, glycerol, or Tris modeled at surface-exposed site on Fe(II)•ADO (PDB: 9DY4); (**D-F**) HYZ, glycerol, and Tris modeled within the active sites of Co(II)•ADO (PDB: 9DMA); (**G-I**) HYZ, glycerol, and Tris modeled at surface-exposed sites on Co(II)•ADO (PDB: 9DMA).

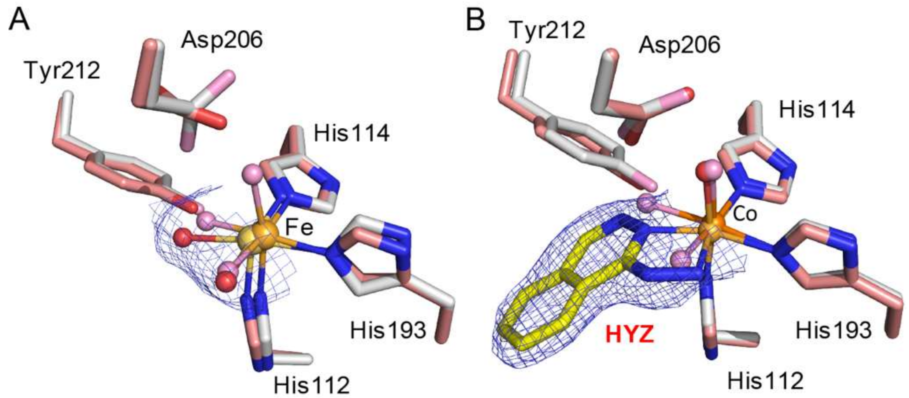

Fig. S10. Structural overlays of HYZ-bound and resting-state ADO. (**A**) Superposition of HYZ-bound Fe(II)•ADO (salmon) with resting-state Fe(II)•ADO (gray); (**B**) HYZ-bound Co(II)•ADO (salmon) with resting-state Co(II)•ADO (gray). 2*Fo*–*Fc* maps are contoured at 1.5 σ and shown in blue. Oxygen atoms from the resting-state ADO are shown in pink; those from the water-bound Fe(II)•ADO and HYZ-bound Co(II)•ADO are shown in red. HYZ is shown in yellow.

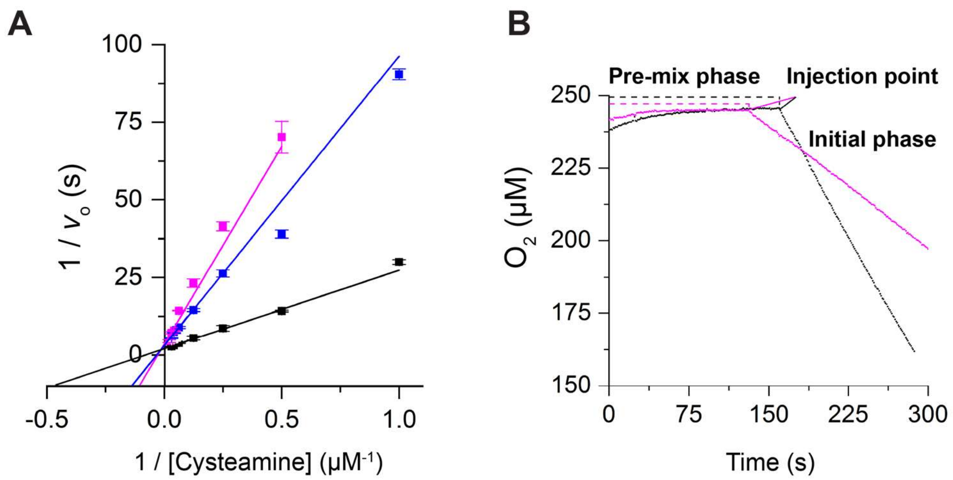

Fig. S11. Inhibition pattern of Fe(II)•ADO by HYZ. (**A**) Lineweaver–Burk plots showing the inhibition of Fe(II)•ADO (2 μM) by HYZ at 0 (black), 6 (blue), and 10 (magenta) μM, based on initial rates of O_2 consumption. Enzyme was pre-incubated with ascorbic acid (20 μM) and the indicated concentrations of HYZ before initiating the reaction with varying concentrations of cysteamine. (**B**) Representative oxygen consumption traces in the presence of 24 mM cysteamine and 10 μM HYZ. The steady O_2 consumption rate following substrate injection indicates that the measured K_i primarily reflects the initial, reversible binding event between HYZ and ADO.

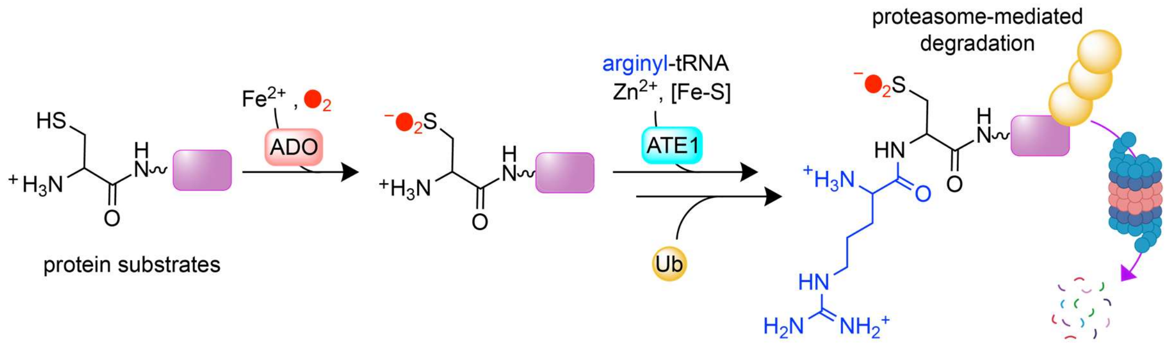

Fig. S12. *N*-degron pathway scheme. *N*-degron pathway initiated by *N*-terminal cysteine oxidation via ADO, followed by *N*-terminal arginylation by arginyltransferase (ATE1). Proteins with post-translationally conjugated *N*-terminal Arg residues can undergo ubiquitination and subsequent proteasome-mediated degradation. Ub: ubiquitin. [Part of figure created with BioRender.com]

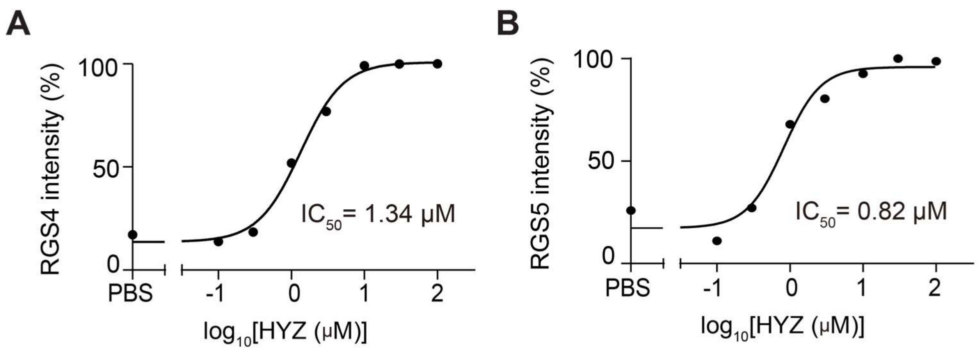

Fig. S13. Western blot-based inhibition curves of ADO by HYZ in SH-SY5Y cells. IC_{50} values were determined by measuring endogenous protein levels of RGS4 (**A**) and RGS5 (**B**) as a function of increasing concentrations of HYZ (1 h treatment).

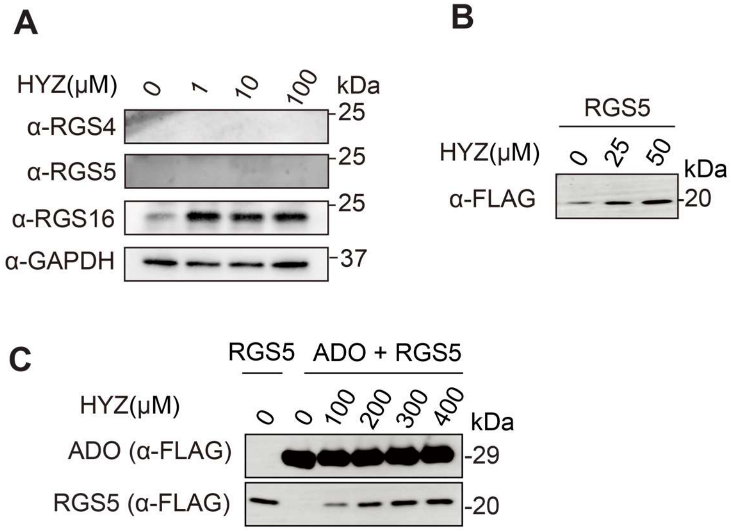

Fig. S14. ADO inhibition in HEK293T cells. Inhibition of ADO activity by HYZ (1 h) in HEK293T cells was quantified with Western blot. (**A**) Endogenous and (**B**) overexpressed RGS protein levels in response to varying concentrations of HYZ. (**C**) Co-overexpressed RGS5 and ADO protein levels in the absence or presence of indicated HYZ concentration

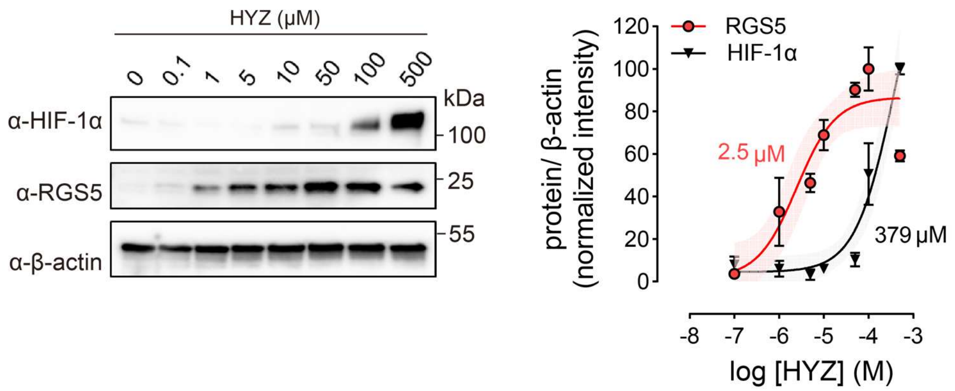

Fig. S15. ADO/RGS5 and PHD/HIF inhibition in SH-SY5Y cells. Endogenous HIF-1α, RGS5 and β-actin protein levels in SH-SY5Y cells treated with varying concentrations of HYZ (4 h). The inhibition curves (right) were determined by the corresponding Western blots (left). Data are represented as mean ± SEM (n = 3 independent experiments).

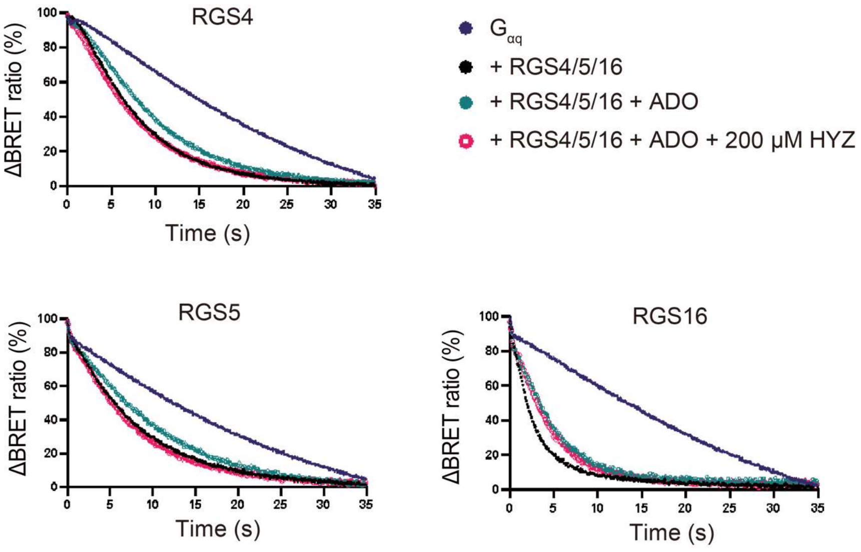

Fig. S16. BRET assay monitoring the RGS-induced acceleration of G protein deactivation. Representative traces showing time-dependent $G_{\alpha q}$ deactivation for cells in the absence (purple) or presence (black) of the indicated RGS expression versus co-expressed with ADO in the absence (green) or presence (red) of HYZ (200 µM, 1 h). k_{GAP} shown in Fig. 4F was calculated from the ΔBRET ratio.

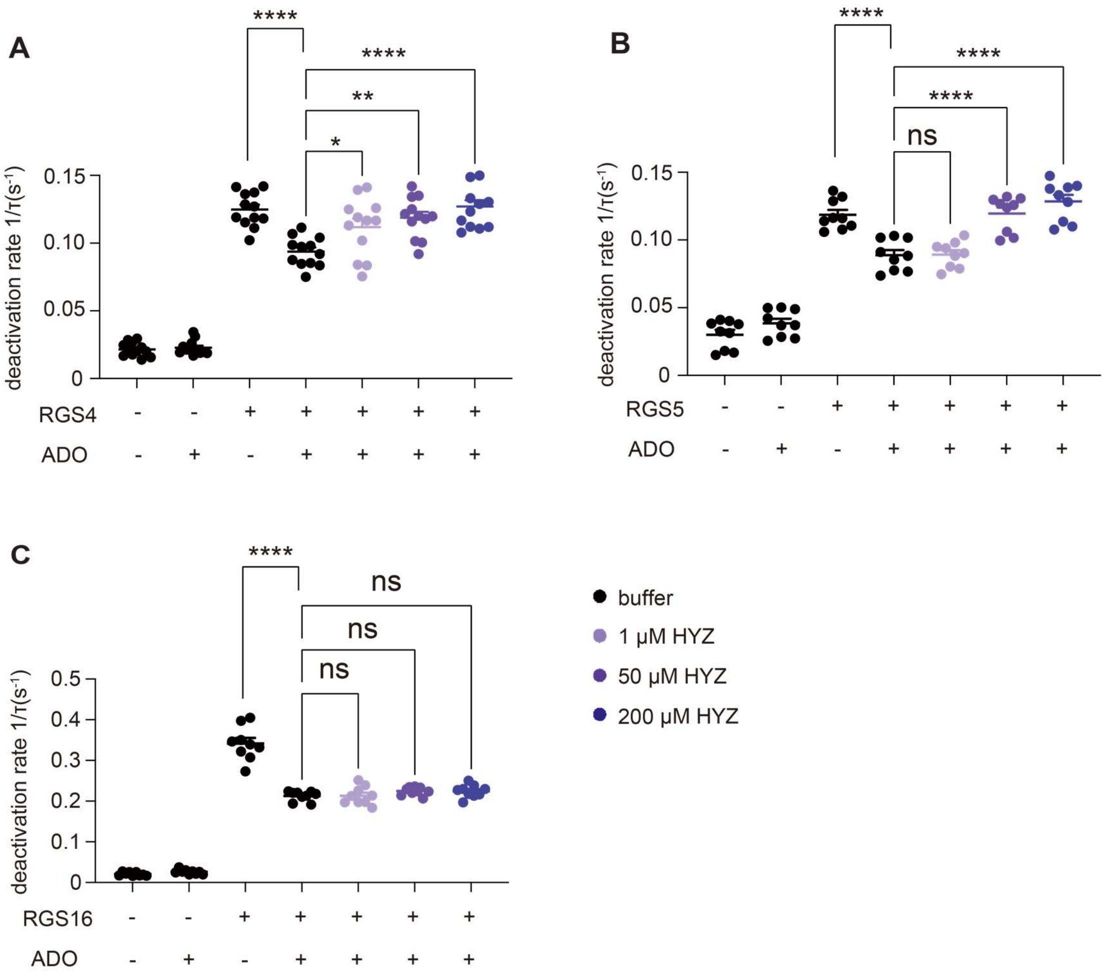

Fig. S17. HYZ restores the GAP activity of RGS4 and RGS5 in a concentration-dependent manner. Overexpression of ADO reduces the GAP activity of RGS4 (**A**), RGS5 (**B**), and RGS16 (**C**). Treatment of HYZ (1 h) reverses the effect of ADO in RGS4- and RGS5-expressing cells. Data are represented as mean ± SEM (n = 3 independent experiments each performed with three replicates). One-way ANOVA with Dunnett's multiple comparisons test (treatments comparison), **p* < 0.05, ***p* < 0.005, *****p* < 0.0001, ns = not significant. Unpaired t-test for comparing cells transfected with RGS only versus with ADO, ****p* < 0.0005, *****p* < 0.0001.

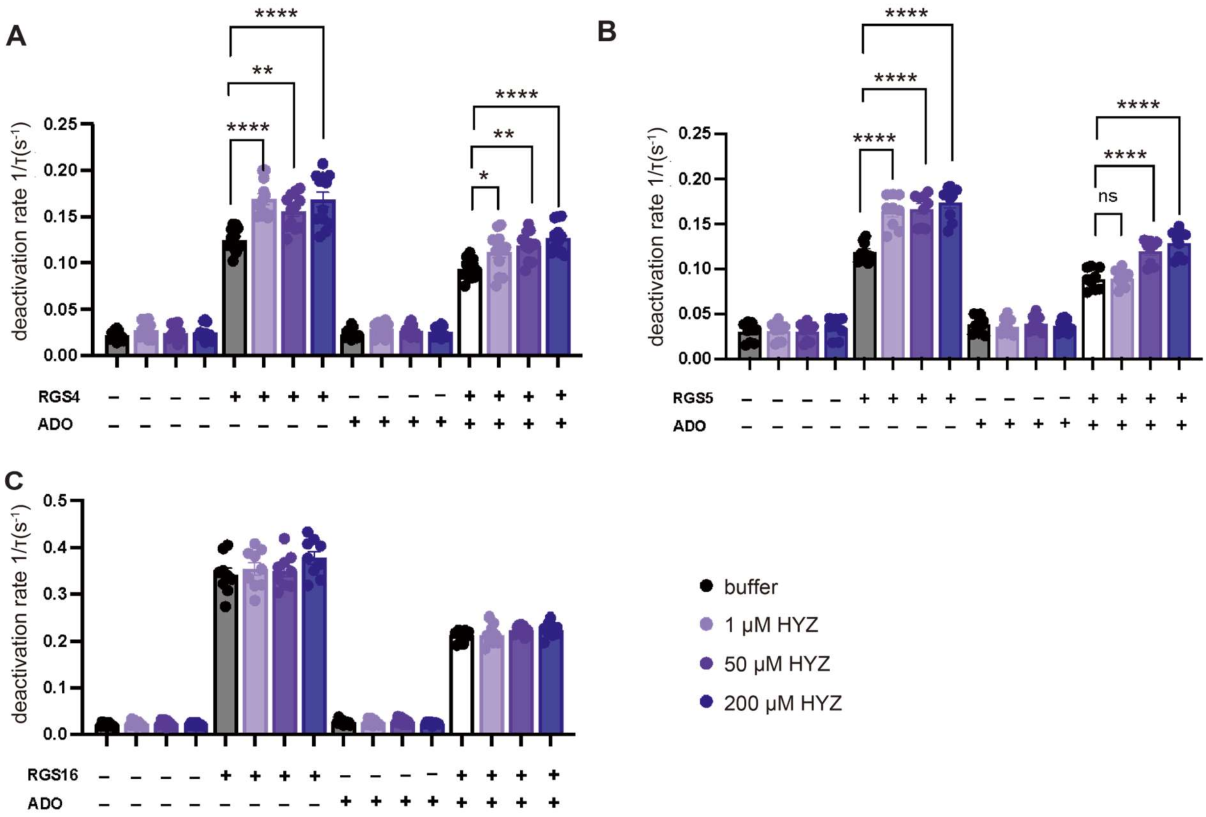

Fig. S18. Summary of the effect of HYZ on deactivation rate for RGS4/5/16. GAP activity was measured in the presence or absence of RGS, ADO, and HYZ. Panels show GAP activity for RGS4 (**A**), RGS5 (**B**), and RGS16 (**C**) treated with 0–200 μM HYZ (1 h). Data are represented as mean ± SEM (n = 3 independent experiments each performed with three replicates). One-way ANOVA with Dunnett's multiple comparisons test, $^{**}p < 0.005$, $^{****}p < 0.0001$.

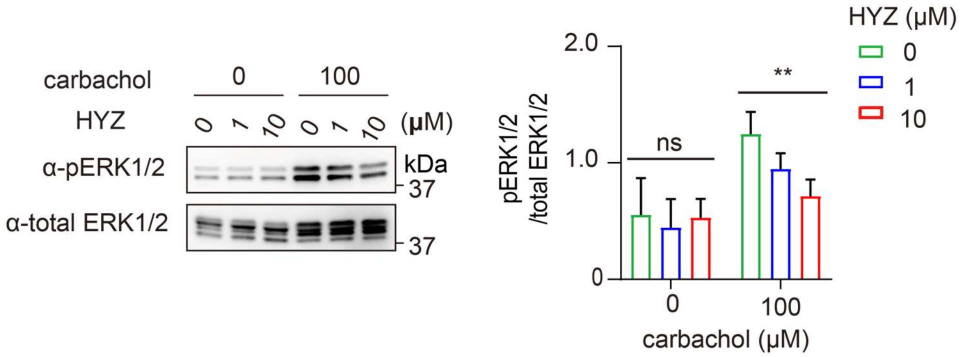

Fig. S19. GPCR-dependent ERK phosphorylation is inhibited by HYZ. Representative Western blot (left) and quantification (right) for total versus phosphorylated ERK1/2 in SH-SY5Y cells following treatment with HYZ (0–100 µM, 1 h). Cells were incubated in the presence or absence of carbachol (100 µM, 5 min) before harvesting. Data are represented as mean ± SD (n = 4 independent experiments). Each data point was compared against a corresponding untreated control with one-way-ANOVA, followed by Tukey's HSD. ***p* < 0.01, ns; not significant.

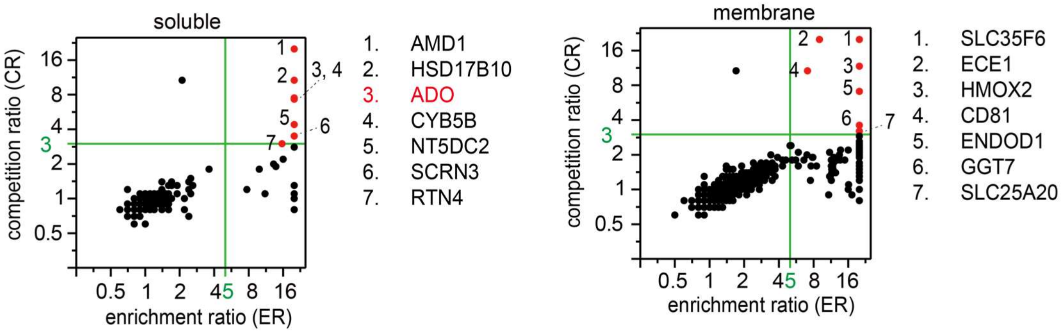

Fig. S20. Identification of HYZyne targets in U-87 cells. Quadrant plot of average enrichment [HYZyne (heavy cells) vs. HYZ (light cells); both treated with 100 µM for 0.5 h] versus competition [HYZyne (heavy cells) vs. HYZyne (light cells) where light cells were first pretreated with 5× HYZ (500 µM; 0.25 h) prior addition of HYZyne (100 µM)]. SILAC protein ratios from quantitative proteomics experiments for soluble and membrane proteomes of U-87 cells are shown. Proteins with ER ≥ 5 and CR ≥ 3 (upper right quadrant) were considered high-occupancy targets; listed to the right of the plot. Proteins highlighted in red are investigated in this work.

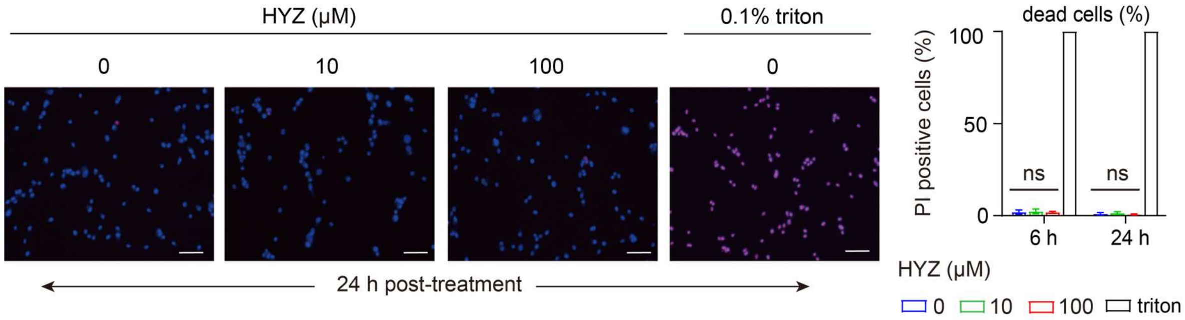

Fig. S21. Cytotoxicity of HYZ treatment. Left: Representative images of Hoechst 33342 (blue) and propidium iodide (PI, red) double staining in U-87 cells following 24 h treatment with HYZ. Bar: 200 μm. Right: percentage of PI positive cells at 6 and 24 h post treatment. Data are represented as mean ± SD (n = 3 independent experiments). Statistical analysis was conducted using one-way ANOVA followed by Tukey's HSD, with each column compared against untreated control cells. The 0.1% triton-treated positive control was excluded from statistical analysis. ns = not significant.

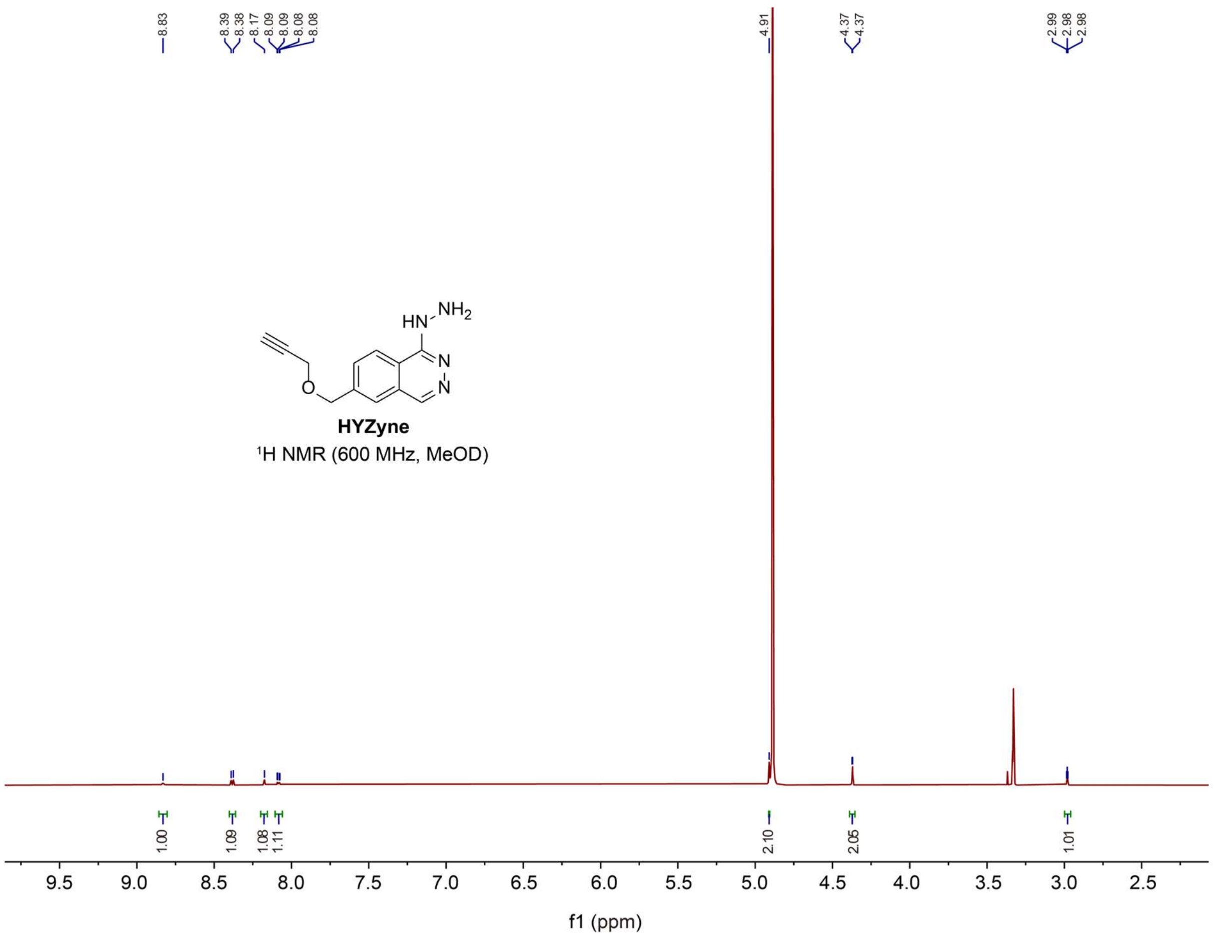

Fig. S22. 1H NMR spectra of HYZyne. 1H NMR spectra of HYZyne hydrochloride salt dissolved in MeOD acquired at 600 MHz.

Table S1. Statistics of monoisotopic masses from MS1 spectra for chemically modified peptides.

protein	*captured peptide*	*tag*	*parent ion intensity*	*charge*	*m/z$_{theor.}$*	*average m/z$_{meas.}$*	*error (ppm)*	*SD*	*n*
ADO	SGTSIPL**H**$_{112}$*DHPGMHGMLK	TEV$_{heavy}$	1.0E+07	3	834.4155	834.4151	-0.4	0.0003	82†
		TEV$_{light}$	8.7E+06	3	832.4109	832.4105	-0.4	0.0004	84†
	QA**C**$_{18}$*LTFR	TEV$_{heavy}$	2.6E+08	3	475.5797	475.5793	-0.7	0.0002	104†
		TEV$_{light}$	2.7E+08	3	473.5751	473.5749	-0.4	0.0003	97†
	IS**C**$_{132}$*MDK	TEV$_{heavy}$	2.7E+07	2	641.8067	641.8065	-0.3	0.0002	43†
		TEV$_{light}$	2.7E+07	2	638.7998	638.7996	-0.2	0.0003	46†
	AEYTEASGP**C**$_{183}$*ILTPHR	TEV$_{heavy}$	1.3E+07	4	583.5387	583.5383	-0.5	0.0007	47†
		TEV$_{light}$	1.3E+07	4	582.0352	582.0349	-0.5	0.0005	44†
	DNLHQIDAVEGPAAFLDILAPPYDPDDGRD**C**$_{220}$*HYYR	TEV$_{heavy}$	7.2E+07	5	912.4318	912.4306	-1.2	0.0009	97†
		TEV$_{light}$	4.1E+07	5	911.2291	911.2281	-1.0	0.0012	93†
	EASSSA**C**$_{249}$*DLPR	TEV$_{heavy}$	2.7E+08	3	574.6067	574.6061	-0.9	0.0004	92†
		TEV$_{light}$	3.7E+08	3	572.6021	572.6017	-0.5	0.0004	91†
	EVWLLETPQADDFW**C**$_{258}$*EPYPGPK	TEV$_{heavy}$	1.0E+07	3	1131.5286	1131.5271	-1.3	0.0009	70†
		TEV$_{light}$	9.7E+07	3	1129.5240	1129.5227	-1.0	0.0012	69†

* site of modification by probe clicked with TEV tags.
† the sum of spectral counts (n ≥ 43) for given peptide from two technical replicates.

Table S2. X-ray crystallographic data collection and refinement statistics

	HYZ bound Co(II)•ADO	Fe(II)•ADO soaked in HYZ
**Data Collection**	SSRL	SSRL
Wavelength (Å)	0.97145	0.97946
Space group	$C222_1$	$C222_1$
Cell dimensions		
a, b, c (Å)	56.3, 95.1, 117.2	56.2, 95.2, 117.8
α, β, γ (°)	90, 90, 90	90, 90, 90
Resolution[a] (Å)	50.00 – 1.89	50.00 – 2.39
	(1.92 – 1.89)	(2.43 – 2.39)
Total reflection	206106	95946
Unique reflection	24951	12657
Redundancy	8.3 (6.4)	7.6 (5.4)
R_{sym} or R_{merge}[b] (%)	10.2 (77.9)	18.1 (80.5)
I/σI	8.1 (1.02)	12.4 (1.04)
Completeness (%)	96.8 (92.3)	98.4 (94.1)
$CC_{1/2}$	0.999 (0.517)	0.951 (0.876)
**Refinement**[c]		
Resolution (Å)	48.47 – 1.88	48.39 – 2.39
No. reflections	24745	12504
R_{work}[d]/R_{free}[e] (%)	21.16 / 25.58	25.25 / 30.76
No. Atoms / *B*-factors (Å^2)		
Protein	1905 / 42.0	1826 / 83.7
Co/Fe	1 / 30.9	1 / 73.2
HYZ	24 / 39.1	12 / 84.9
Glycerol	18 / 51.6	12 / 90.6
Sulfate	5 / 72.6	N/A
Water	100 / 41.9	19 / 85.0
r.m.s. deviations		
Bond lengths[d] (Å)	0.006	0.008
Bond angles (°)	0.867	1.065
Ramachandran[e]		
Favored (%)	98.71	97.3
Allowed (%)	1.29	2.20
Outlier (%)	0.00	0.50
**PDB Entry Code**	**9DMA**	**9DY4**

[a] Values in parentheses are for the highest resolution shell.

[b] $R_{merge} = \Sigma_{hkl}\Sigma i \; |I_i(hkl) - \langle I(hkl)\rangle| / \Sigma_{hkl}\Sigma_i I_i(hkl)$, in which the sum is over all the *I* measured reflections with equivalent miller indices *hkl*; $\langle I(hkl)\rangle$ is the averaged intensity of these *i* reflections, and the grand sum is over all measured reflections in the data set.

[c] All positive reflections were used in the refinement.

[d] According to previous literature (*78*).

[e] Ramachandran statistics were analyzed using MolProbity (*79*). The outlier in 9DY4 is Pro115.

Table S3. Comparison of the EPR parameters for Co(II)•ADO, Co(II)•ADO (cysteamine), and Co(II)•ADO (HYZ).

Sample	*S*	*g* values (g_x, g_y, g_z)
Co(II)•ADO (*26*)	3/2	2.63, 4.25, 5.26
Co(II)•ADO (cysteamine) (*26*)	3/2	2.25, 4.00, 5.71
Co(II)•ADO (HYZ)	3/2	2.55, 4.41, 5.40

Data S1. Compiled average SILAC protein ratios with standard deviation for enrichment and competition experiments with HYZyne for HEK293T and U-87 cell lines followed by representative datasets for each type of experiment. These datasets are shown in separate tabs (8 in total), each displaying median SILAC ratios for all quantified tryptic peptides per protein. Corresponding peptide sequences, masses, charge states, and individual SILAC ratios are shown.

Data S2. Compiled average SILAC and ReDiMe protein ratios with standard deviation for enrichment and competition experiments with HYZyne for RAW264.7 and Neuro 2a cell lines, and enrichment experiments with mouse heart and brain followed by representative datasets for each type of experiment. These datasets are shown in separate tabs (12 in total), each displaying median SILAC or ReDiMe ratios for all quantified tryptic peptides per protein. Corresponding peptide sequences, masses, charge states, and individual SILAC or ReDiMe ratios are shown.

Data S3. Primers used for qPCR.

REFERENCES AND NOTES

1. J. G. Lombardino, J. A. Lowe III, The role of the medicinal chemist in drug discover — Then and now. *Nat. Rev. Drug Discov.* **3**, 853–862 (2004).

2. M. M. Reidenberg, World Health Organization program for the selection and use of essential medicines. *Clin. Pharmacol. Ther.* **81**, 603–606 (2007).

3. L. L. Herman, Z. S. Bruss, V. S. Tivakaran, "Hydralazine," in *StatPearls* (Treasure Island, 2023).

4. S. Donel, D. A. Novri, Y. Hamidy, M. Savira, Effectiveness of nifedipine, labetalol, and hydralazine as emergency antihypertension in severe preeclampsia: A randomized control trial. *F1000Res* **11**, 1287 (2022).

5. M. N. McComb, J. Y. Chao, T. M. Ng, Direct vasodilators and sympatholytic agents. *J. Cardiovasc. Pharmacol. Ther.* **21**, 3–19 (2016).

6. H. J. Knowles, Y. M. Tian, D. R. Mole, A. L. Harris, Novel mechanism of action for hydralazine: Induction of hypoxia-inducible factor-1α, vascular endothelial growth factor, and angiogenesis by inhibition of prolyl hydroxylases. *Circ. Res.* **95**, 162–169 (2004).

7. E. Dehghan, Y. Zhang, B. Saremi, S. Yadavali, A. Hakimi, M. Dehghani, M. Goodarzi, X. Tu, S. Robertson, R. Lin, A. Chudhuri, H. Mirzaei, Hydralazine induces stress resistance and extends *C. elegans* lifespan by activating the NRF2/SKN-1 signalling pathway. *Nat. Commun.* **8**, 2223 (2017).

8. J. P. Barnes, S. M. Yang, T. S. Thompson, C. M. Guevarra, S. S. Pleasant, T. Q. Do, R. D. Crouch, Mechanistic investigation of the time-dependent aldehyde oxidase inhibitor hydralazine. *Drug Metab. Dispos.* **51**, 782–791 (2023).

9. E. Dehghan, M. Goodarzi, B. Saremi, R. Lin, H. Mirzaei, Hydralazine targets cAMP-dependent protein kinase leading to sirtuin1/5 activation and lifespan extension in *C. elegans*. *Nat. Commun.* **10**, 4905 (2019).

10. Q. Wu, Z. Sun, Z. Chen, J. Liu, H. Ding, C. Luo, M. Wang, D. Du, The discovery of a non-competitive GOT1 inhibitor, hydralazine hydrochloride, via a coupling reaction-based high-throughput screening assay. *Bioorg. Med. Chem. Lett.* **73**, 128883 (2022).

11. M. L. Matthews, L. He, B. D. Horning, E. J. Olson, B. E. Correia, J. R. Yates III, P. E. Dawson, B. F. Cravatt, Chemoproteomic profiling and discovery of protein electrophiles in human cells. *Nat. Chem.* **9**, 234–243 (2017).

12. Z. Lin, X. Wang, K. A. Bustin, K. Shishikura, N. R. McKnight, L. He, R. M. Suciu, K. Hu, X. Han, M. Ahmadi, E. J. Olson, W. H. Parsons, M. L. Matthews, Activity-based hydrazine probes for protein profiling of electrophilic functionality in therapeutic targets. *ACS Cent. Sci.* **7**, 1524–1534 (2021).

13. X. Wang, Z. Lin, K. A. Bustin, N. R. McKnight, W. H. Parsons, M. L. Matthews, Discovery of potent and selective inhibitors against protein-derived electrophilic cofactors. *J. Am. Chem. Soc.* **144**, 5377–5388 (2022).

14. K. A. Bustin, A. Abbas, X. Wang, M. C. Abt, J. P. Zackular, M. L. Matthews, Characterizing metabolic drivers of *Clostridioides difficile* infection with activity-based hydrazine probes. *Front. Pharmacol.* **14**, 1074619 (2023).

15. K. A. Bustin, K. Shishikura, I. Chen, Z. Lin, N. McKnight, Y. Chang, X. Wang, J. J. Li, E. Arellano, L. Pei, P. D. Morton, A. M. Gregus, M. W. Buczynski, M. L. Matthews, Phenelzine-based probes reveal Secernin-3 is involved in thermal nociception. *Mol. Cell. Neurosci.* **125**, 103842 (2023).

16. B. F. Cravatt, A. T. Wright, J. W. Kozarich, Activity-based protein profiling: From enzyme chemistry to proteomic chemistry. *Annu. Rev. Biochem.* **77**, 383–414 (2008).

17. J. E. Dominy Jr., C. R. Simmons, L. L. Hirschberger, J. Hwang, R. M. Coloso, M. H. Stipanuk, Discovery and characterization of a second mammalian thiol dioxygenase, cysteamine dioxygenase. *J. Biol. Chem.* **282**, 25189–25198 (2007).

18. N. Masson, T. P. Keeley, B. Giuntoli, M. D. White, M. L. Puerta, P. Perata, R. J. Hopkinson, E. Flashman, F. Licausi, P. J. Ratcliffe, Conserved N-terminal cysteine dioxygenases transduce responses to hypoxia in animals and plants. *Science* **365**, 65–69 (2019).

19. Y. Wang, O. Sargisson, D. T. Nguyen, K. Parker, S. J. R. Pyke, A. Alramahi, L. Thihlum, Y. Fang, M. E. Wallace, S. P. Berzins, E. Oqueli, D. J. Magliano, J. Golledge, Effect of hydralazine on angiotensin II-induced abdominal aortic aneurysm in apolipoprotein E-deficient mice. *Int. J. Mol. Sci.* **24**, 15955 (2023).

20. P. J. Boersema, R. Raijmakers, S. Lemeer, S. Mohammed, A. J. Heck, Multiplex peptide stable isotope dimethyl labeling for quantitative proteomics. *Nat. Protoc.* **4**, 484–494 (2009).

21. C. Liu, A. T. Kraja, J. A. Smith, J. A. Brody, N. Franceschini, J. C. Bis, K. Rice, A. C. Morrison, Y. Lu, S. Weiss, X. Guo, W. Palmas, L. W. Martin, Y. D. Chen, P. Surendran, F. Drenos, J. P. Cook, P. L. Auer, A. Y. Chu, A. Giri, W. Zhao, J. Jakobsdottir, L. A. Lin, J. M. Stafford, N. Amin, H. Mei, J. Yao, A. Voorman, CHD Exome+ Consortium, ExomeBP Consortium, GoT2DGenes Consortium, T2D-GENES Consortium, M. G. Larson, M. L. Grove, A. V. Smith, S. J. Hwang, H. Chen, T. Huan, G. Kosova, N. O. Stitziel, S. Kathiresan, N. Samani, H. Schunkert, P. Deloukas, Myocardial Infarction Genetics and CARDIoGRAM Exome Consortia, M. Li, C. Fuchsberger, C. Pattaro, M. Gorski, CKDGen Consortium, C. Kooperberg, G. J. Papanicolaou, J. E. Rossouw, J. D. Faul, S. L. Kardia, C. Bouchard, L. J. Raffel, A. G. Uitterlinden, O. H. Franco, R. S. Vasan, C. J. O'Donnell, K. D. Taylor, K. Liu, E. P. Bottinger, O. Gottesman, E. W. Daw, F. Giulianini, S. Ganesh, E. Salfati, T. B. Harris, L. J. Launer, M. Dorr, S. B. Felix, R. Rettig, H. Volzke, E. Kim, W. J. Lee, I. T. Lee, W. H. Sheu, K. S. Tsosie, D. R. Edwards, Y. Liu, A. Correa, D. R. Weir, U. Volker, P. M. Ridker, E. Boerwinkle, V. Gudnason, A. P. Reiner, C. M. van Duijn, I. B. Borecki, T. L. Edwards, A. Chakravarti, J. I. Rotter, B. M. Psaty, R. J. Loos, M. Fornage, G. B. Ehret, C. Newton-Cheh, D. Levy, D. I. Chasman, Meta-analysis identifies common and rare variants influencing blood pressure and overlapping with metabolic trait loci. *Nat. Genet.* **48**, 1162–1170 (2016).

22. P. Surendran, F. Drenos, R. Young, H. Warren, J. P. Cook, A. K. Manning, N. Grarup, X. Sim, D. R. Barnes, K. Witkowska, J. R. Staley, V. Tragante, T. Tukiainen, H. Yaghootkar, N. Masca, D. F. Freitag, T. Ferreira, O. Giannakopoulou, A. Tinker, M. Harakalova, E. Mihailov,

C. Liu, A. T. Kraja, S. F. Nielsen, A. Rasheed, M. Samuel, W. Zhao, L. L. Bonnycastle, A. U. Jackson, N. Narisu, A. J. Swift, L. Southam, J. Marten, J. R. Huyghe, A. Stancakova, C. Fava, T. Ohlsson, A. Matchan, K. E. Stirrups, J. Bork-Jensen, A. P. Gjesing, J. Kontto, M. Perola, S. Shaw-Hawkins, A. S. Havulinna, H. Zhang, L. A. Donnelly, C. J. Groves, N. W. Rayner, M. J. Neville, N. R. Robertson, A. M. Yiorkas, K. H. Herzig, E. Kajantie, W. Zhang, S. M. Willems, L. Lannfelt, G. Malerba, N. Soranzo, E. Trabetti, N. Verweij, E. Evangelou, A. Moayyeri, A. C. Vergnaud, C. P. Nelson, A. Poveda, T. V. Varga, M. Caslake, A. J. de Craen, S. Trompet, J. Luan, R. A. Scott, S. E. Harris, D. C. Liewald, R. Marioni, C. Menni, A. E. Farmaki, G. Hallmans, F. Renstrom, J. E. Huffman, M. Hassinen, S. Burgess, R. S. Vasan, J. F. Felix, CHARGE Heart Failure Consortium, M. Uria-Nickelsen, A. Malarstig, D. F. Reily, M. Hoek, T. Vogt, H. Lin, W. Lieb, EchoGen Consortium, M. Traylor, H. F. Markus, METASTROKE Consortium, H. M. Highland, A. E. Justice, E. Marouli, GIANT Consortium, J. Lindstrom, M. Uusitupa, P. Komulainen, T. A. Lakka, R. Rauramaa, O. Polasek, I. Rudan, O. Rolandsson, P. W. Franks, G. Dedoussis, T. D. Spector, EPIC-InterAct Consortium, P. Jousilahti, S. Mannisto, I. J. Deary, J. M. Starr, C. Langenberg, N. J. Wareham, M. J. Brown, A. F. Dominiczak, J. M. Connell, J. W. Jukema, N. Sattar, I. Ford, C. J. Packard, T. Esko, R. Magi, A. Metspalu, R. A. de Boer, P. van der Meer, P. van der Harst, S. L. Cohort, G. Gambaro, E. Ingelsson, L. Lind, P. I. de Bakker, M. E. Numans, I. Brandslund, C. Christensen, E. R. Petersen, E. Korpi-Hyovalti, H. Oksa, J. C. Chambers, J. S. Kooner, A. I. Blakemore, S. Franks, M. R. Jarvelin, L. L. Husemoen, A. Linneberg, T. Skaaby, B. Thuesen, F. Karpe, J. Tuomilehto, A. S. Doney, A. D. Morris, C. N. Palmer, O. L. Holmen, K. Hveem, C. J. Willer, T. Tuomi, L. Groop, A. Karajamaki, A. Palotie, S. Ripatti, V. Salomaa, D. S. Alam, A. A. S. Majumder, E. Di Angelantonio, R. Chowdhury, M. I. McCarthy, N. Poulter, A. V. Stanton, P. Sever, P. Amouyel, D. Arveiler, S. Blankenberg, J. Ferrieres, F. Kee, K. Kuulasmaa, M. Muller-Nurasyid, G. Veronesi, J. Virtamo, P. Deloukas, Wellcome Trust Case Control Consortium, P. Elliott, Understanding Society Scientific Group, E. Zeggini, S. Kathiresan, O. Melander, J. Kuusisto, M. Laakso, S. Padmanabhan, D. Porteous, C. Hayward, G. Scotland, F. S. Collins, K. L. Mohlke, T. Hansen, O. Pedersen, M. Boehnke, H. M. Stringham, EPIC-CVD Consortium, P. Frossard, C. Newton-Cheh, CHARGE+ Exome Chip Blood Pressure Consortium, M. D. Tobin, B. G. Nordestgaard, T2D-GENES Consortium, GoT2DGenes Consortium, ExomeBP Consortium, CHD Exome+ Consortium, M. J. Caulfield, A. Mahajan, A. P. Morris, M. Tomaszewski, N. J. Samani, D.

Saleheen, F. W. Asselbergs, C. M. Lindgren, J. Danesh, L. V. Wain, A. S. Butterworth, J. M. Howson, P. B. Munroe, Trans-ancestry meta-analyses identify rare and common variants associated with blood pressure and hypertension. *Nat. Genet.* **48**, 1151–1161 (2016).

23. E. Weerapana, A. E. Speers, B. F. Cravatt, Tandem orthogonal proteolysis-activity-based protein profiling (TOP-ABPP)—A general method for mapping sites of probe modification in proteomes. *Nat. Protoc.* **2**, 1414–1425 (2007).

24. M. D. White, L. Dalle Carbonare, M. Lavilla Puerta, S. Iacopino, M. Edwards, K. Dunne, E. Pires, C. Levy, M. A. McDonough, F. Licausi, E. Flashman, Structures of *Arabidopsis thaliana* oxygen-sensing plant cysteine oxidases 4 and 5 enable targeted manipulation of their activity. *Proc. Natl. Acad. Sci. U.S.A.* **117**, 23140–23147 (2020).

25. Y. Wang, I. Shin, J. Li, A. Liu, Crystal structure of human cysteamine dioxygenase provides a structural rationale for its function as an oxygen sensor. *J. Biol. Chem.* **297**, 101176 (2021).

26. J. Li, R. Duan, A. Liu, Cobalt(II)-substituted cysteamine dioxygenase oxygenation proceeds through a cobalt(III)-superoxo complex. *J. Am. Chem. Soc.* **146**, 18292–18297 (2024).

27. Y. M. Tian, P. Holdship, T. Q. To, P. J. Ratcliffe, T. P. Keeley, Comparative analysis of N-terminal cysteine dioxygenation and prolyl-hydroxylation as oxygen-sensing pathways in mammalian cells. *J. Biol. Chem.* **299**, 105156 (2023).

28. S. C. S. Lee, A. H. A. Pyo, M. Koritzinsky, Longitudinal dynamics of the tumor hypoxia response: From enzyme activity to biological phenotype. *Sci. Adv.* **9**, eadj6409 (2023).

29. Y. Wang, I. Davis, Y. Chan, S. G. Naik, W. P. Griffith, A. Liu, Characterization of the nonheme iron center of cysteamine dioxygenase and its interaction with substrates. *J. Biol. Chem.* **295**, 11789–11802 (2020).

30. R. A. Copeland, "Irreversible enzyme inactivators", in *Evaluation of Enzyme Enhibitors in Drug Discovery* (John Wiley & Sons, 2013), pp. 345–382.

31. A. Varshavsky, N-degron and C-degron pathways of protein degradation. *Proc. Natl. Acad. Sci. U.S.A.* **116**, 358–366 (2019).

32. E. M. Ross, T. M. Wilkie, GTPase-activating proteins for heterotrimeric G proteins: Regulators of G protein signaling (RGS) and RGS-like proteins. *Annu. Rev. Biochem.* **69**, 795–827 (2000).

33. M. J. Lee, T. Tasaki, K. Moroi, J. Y. An, S. Kimura, I. V. Davydov, Y. T. Kwon, RGS4 and RGS5 are in vivo substrates of the N-end rule pathway. *Proc. Natl. Acad. Sci. U.S.A.* **102**, 15030–15035 (2005).

34. S. M. Shim, H. R. Choi, S. C. Kwon, H. Y. Kim, K. W. Sung, E. J. Jung, S. R. Mun, T. H. Bae, D. H. Kim, Y. S. Son, C. H. Jung, J. Lee, M. J. Lee, J. W. Park, Y. T. Kwon, The Cys-N-degron pathway modulates pexophagy through the N-terminal oxidation and arginylation of ACAD10. *Autophagy* **19**, 1642–1661 (2023).

35. A. Siedlecki, J. R. Anderson, X. Jin, J. R. Garbow, T. S. Lupu, A. J. Muslin, RGS4 controls renal blood flow and inhibits cyclosporine-mediated nephrotoxicity. *Am. J. Transplant.* **10**, 231–241 (2010).

36. V. Holobotovskyy, Y. S. Chong, J. Burchell, B. He, M. Phillips, L. Leader, T. V. Murphy, S. L. Sandow, D. J. McKitrick, A. K. Charles, M. Tare, L. F. Arnolda, R. Ganss, Regulator of G protein signaling 5 is a determinant of gestational hypertension and preeclampsia. *Sci. Transl. Med.* **7**, 290ra288 (2015).

37. I. Masuho, S. Balaji, B. S. Muntean, N. K. Skamangas, S. Chavali, J. J. G. Tesmer, M. M. Babu, K. A. Martemyanov, A global map of G protein signaling regulation by RGS proteins. *Cell* **183**, 503–521.e9 (2020).

38. D. G. Lambert, A. S. Ghataorre, S. R. Nahorski, Muscarinic receptor binding characteristics of a human neuroblastoma SK-N-SH and its clones SH-SY5Y and SH-EP1. *Eur. J. Pharmacol.* **165**, 71–77 (1989).

39. C. D. Vedove, M. Del Giglio, D. Schena, G. Girolomoni, Drug-induced lupus erythematosus. *Arch. Dermatol. Res.* **301**, 99–105 (2009).

40. R. Yung, S. Chang, N. Hemati, K. Johnson, B. Richardson, Mechanisms of drug-induced lupus. IV. Comparison of procainamide and hydralazine with analogs in vitro and in vivo. *Arthritis Rheumatol.* **40**, 1436–1443 (1997).

41. J. Quddus, K. J. Johnson, J. Gavalchin, E. P. Amento, C. E. Chrisp, R. L. Yung, B. C. Richardson, Treating activated CD4^{+} T cells with either of two distinct DNA methyltransferase inhibitors, 5-azacytidine or procainamide, is sufficient to cause a lupus-like disease in syngeneic mice. *J. Clin. Invest.* **92**, 38–53 (1993).

42. A. H. Sawalha, Editorial: The innate and adaptive immune response are both involved in drug-induced autoimmunity. *Arthritis Rheumatol.* **70**, 330–333 (2018).

43. R. Jain, U. Watson, L. Vasudevan, D. K. Saini, ERK activation pathways downstream of GPCRs. *Int. Rev. Cell Mol. Biol.* **338**, 79–109 (2018).

44. P. Gao, C. Yang, C. L. Nesvick, M. J. Feldman, S. Sizdahkhani, H. Liu, H. Chu, F. Yang, L. Tang, J. Tian, S. Zhao, G. Li, J. D. Heiss, Y. Liu, Z. Zhuang, G. Xu, Hypotaurine evokes a malignant phenotype in glioma through aberrant hypoxic signaling. *Oncotarget* **7**, 15200–15214 (2016).

45. D. Shen, L. Tian, F. Yang, J. Li, X. Li, Y. Yao, E. W. Lam, P. Gao, B. Jin, R. Wang, ADO/hypotaurine: A novel metabolic pathway contributing to glioblastoma development. *Cell Death Discov.* **7**, 21 (2021).

46. Cancer Genome Atlas Research Network, J. N. Weinstein, E. A. Collisson, G. B. Mills, K. R. Shaw, B. A. Ozenberger, K. Ellrott, I. Shmulevich, C. Sander, J. M. Stuart, The cancer genome atlas pan-cancer analysis project. *Nat. Genet.* **45**, 1113–1120 (2013).

47. S. C. S. Lee, A. H. A. Pyo, H. Mohammadi, J. Zhang, A. Dvorkin-Gheva, L. Malbeteau, S. Chung, S. Khan, M. T. Ciudad, V. Rondeau, R. A. Cairns, T. Kislinger, T. L. McGaha, B. G. Wouters, J. A. Reisz, R. Culp-Hill, A. D’Alessandro, C. L. Jones, M. Koritzinsky, Cysteamine dioxygenase (ADO) governs cancer cell mitochondrial redox homeostasis through proline metabolism. *Sci. Adv.* **10**, eadq0355 (2024).

48. O. Rixe, T. Fojo, Is cell death a critical end point for anticancer therapies or is cytostasis sufficient? *Clin. Cancer Res.* **13**, 7280–7287 (2007).

49. H. Cao, C. Li, W. Qi, X. Meng, R. Tian, Y. Qi, W. Yang, J. Li, Synthesis, cytotoxicity and antitumour mechanism investigations of polyoxometalate doped silica nanospheres on breast cancer MCF-7 cells. *PLOS ONE* **12**, e0181018 (2017).

50. A. Y. Oral, H. B. Oral, M. Sarimahmut, B. Cevatemre, G. Ozkaya, Ş. Korkmaz, E. Ulukaya, Combination of esomeprazole with chemotherapeutics results in more pronounced cytotoxic effect via apoptosis on A549 nonsmall-cell lung cancer cell line. *Turk. J. Biol.* **41**, 231–241 (2017).

51. C. A. Schmitt, B. Wang, M. Demaria, Senescence and cancer — Role and therapeutic opportunities. *Nat. Rev. Clin. Oncol.* **19**, 619–636 (2022).

52. X. Wang, K. C. Yip, A. He, J. Tang, S. Liu, R. Yan, Q. Zhang, R. Li, Plasma olink proteomics identifies CCL20 as a novel predictive and diagnostic inflammatory marker for preeclampsia. *J. Proteome Res.* **21**, 2998–3006 (2022).

53. M. Maheshwari, J. K. Roberts, B. Desutter, K. T. Duong, J. Tingling, J. N. Fawver, H. E. Schall, M. Kahle, I. V. Murray, Hydralazine modifies Aβ fibril formation and prevents modification by lipids in vitro. *Biochemistry* **49**, 10371–10380 (2010).

54. M. Chatard, C. Puech, F. Roche, N. Perek, Hypoxic stress induced by hydralazine leads to a loss of blood-brain barrier integrity and an increase in efflux transporter activity. *PLOS ONE* **11**, e0158010 (2016).

55. M. V. S. Maturu, S. Pappu, A. V. Datla, A. Devara, S. Dalai, Atypical presentation of antenatal eclampsia. *Cureus* **14**, e24745 (2022).

56. N. Sladojevic, B. Yu, J. K. Liao, Regulator of G-protein signaling 5 maintains brain endothelial cell function in focal cerebral ischemia. *J. Am. Heart Assoc.* **9**, e017533 (2020).

57. N. H. Harbin, D. J. Lustberg, C. Hurst, J. Pare, K. M. Crotty, A. L. Waters, S. M. Yeligar, Y. Smith, N. T. Seyfried, D. Weinshenker, J. R. Hepler, RGS14 limits seizure-induced

mitochondrial oxidative stress and pathology in hippocampus. *Neurobiol. Dis.* **181**, 106128 (2023).

58. H. Feng, B. Sjogren, B. Karaj, V. Shaw, A. Gezer, R. R. Neubig, Movement disorder in GNAO1 encephalopathy associated with gain-of-function mutations. *Neurology* **89**, 762–770 (2017).

59. L. A. Magee, K. H. Nicolaides, P. von Dadelszen, Preeclampsia. *N. Engl. J. Med.* **386**, 1817–1832 (2022).

60. C. Trautwein, L. Zizmare, I. Maurer, B. Bender, B. Bayer, U. Ernemann, M. Tatagiba, S. J. Grau, B. J. Pichler, M. Skardelly, G. Tabatabai, Tissue metabolites in diffuse glioma and their modulations by IDH1 mutation, histology, and treatment. *JCI Insight* **7**, e153526 (2022).

61. J. Cornish, K. E. Callon, C. Q. Lin, C. L. Xiao, T. B. Mulvey, G. J. Cooper, I. R. Reid, Trifluoroacetate, a contaminant in purified proteins, inhibits proliferation of osteoblasts and chondrocytes. *Am. J. Physiol.* **277**, E779–E783 (1999).

62. W. Dekant, R. Dekant, Mammalian toxicity of trifluoroacetate and assessment of human health risks due to environmental exposures. *Arch. Toxicol.* **97**, 1069–1077 (2023).

63. M. P. Washburn, D. Wolters, J. R. Yates III, Large-scale analysis of the yeast proteome by multidimensional protein identification technology. *Nat. Biotechnol.* **19**, 242–247 (2001).

64. M. Mann, Functional and quantitative proteomics using SILAC. *Nat. Rev. Mol. Cell Biol.* **7**, 952–958 (2006).

65. S. C. Gill, P. H. von Hippel, Calculation of protein extinction coefficients from amino acid sequence data. *Anal. Biochem.* **182**, 319–326 (1989).

66. Z. Otwinowski, W. Minor, Processing of x-ray diffraction data collected in oscillation mode. *Methods Enzymol.* **276**, 307–326 (1997).

67. P. D. Adams, P. V. Afonine, G. Bunkoczi, V. B. Chen, I. W. Davis, N. Echols, J. J. Headd, L. W. Hung, G. J. Kapral, R. W. Grosse-Kunstleve, A. J. McCoy, N. W. Moriarty, R. Oeffner, R.

J. Read, D. C. Richardson, J. S. Richardson, T. C. Terwilliger, P. H. Zwart, PHENIX: A comprehensive Python-based system for macromolecular structure solution. *Acta Crystallogr. D Biol. Crystallogr.* **66**, 213–221 (2010).

68. P. Emsley, K. Cowtan, Coot: Model-building tools for molecular graphics. *Acta Crystallogr. D Biol. Crystallogr.* **60**, 2126–2132 (2004).

69. R. Duan, J. Li, A. Liu, "Chapter Seven - Unveiling the mechanism of cysteamine dioxygenase: A combined HPLC-MS assay and metal-substitution approach," in *Methods in Enzymology*, J. Bridwell-Rabb, Ed. (Academic Press, 2024), vol. 703, pp. 147–166.

70. M. J. Niphakis, B. F. Cravatt, Enzyme inhibitor discovery by activity-based protein profiling. *Annu. Rev. Biochem.* **83**, 341–377 (2014).

71. S. Wang, Y. Tian, M. Wang, M. Wang, G. B. Sun, X. B. Sun, Advanced activity-based protein profiling application strategies for drug development. *Front. Pharmacol.* **9**, 353 (2018).

72. I. Masuho, O. Ostrovskaya, G. M. Kramer, C. D. Jones, K. Xie, K. A. Martemyanov, Distinct profiles of functional discrimination among G proteins determine the actions of G protein-coupled receptors. *Sci. Signal.* **8**, ra123 (2015).

73. S. Al-Nasiry, N. Geusens, M. Hanssens, C. Luyten, R. Pijnenborg, The use of Alamar Blue assay for quantitative analysis of viability, migration and invasion of choriocarcinoma cells. *Hum. Reprod.* **22**, 1304–1309 (2007).

74. T. D. Schmittgen, K. J. Livak, Analyzing real-time PCR data by the comparative C_T method. *Nat. Protoc.* **3**, 1101–1108 (2008).

75. F. Li, P. Liu, W. Mi, L. Li, N. M. Anderson, N. P. Lesner, M. Burrows, J. Plesset, A. Majer, G. Wang, J. Li, L. Zhu, B. Keith, M. C. Simon, Blocking methionine catabolism induces senescence and confers vulnerability to GSK3 inhibition in liver cancer. *Nat. Cancer* **5**, 131–146 (2024).

76. K. Shishikura, T. Horiuchi, N. Sakata, D. A. Trinh, R. Shirakawa, T. Kimura, Y. Asada, H. Horiuchi, Prostaglandin E2 inhibits neutrophil extracellular trap formation through production of cyclic AMP. *Br. J. Pharmacol.* **173**, 319–331 (2016).

77. M. W. Buczynski, M. A. Herman, K. L. Hsu, L. A. Natividad, C. Irimia, I. Y. Polis, H. Pugh, J. W. Chang, M. J. Niphakis, B. F. Cravatt, M. Roberto, L. H. Parsons, Diacylglycerol lipase disinhibits VTA dopamine neurons during chronic nicotine exposure. *Proc. Natl. Acad. Sci. U.S.A.* **113**, 1086–1091 (2016).

78. R. A. Engh, R. Huber, Accurate bond and angle parameters for x-ray protein structure refinement. *Acta Crystallogr. Sect. A. Found. Crystallogr.* **47**, 392–400 (1991).

79. S. C. Lovell, I. W. Davis, W. B. Arendall III, P. I. de Bakker, J. M. Word, M. G. Prisant, J. S. Richardson, D. C. Richardson, Structure validation by Cα geometry: ϕ,ψ and Cβ deviation. *Proteins* **50**, 437–450 (2003).
